# Social media use and mental health during the COVID-19 pandemic in young adults: a meta-analysis of 14 cross-sectional studies

**DOI:** 10.1186/s12889-022-13409-0

**Published:** 2022-05-17

**Authors:** Youngrong Lee, Ye Jin Jeon, Sunghyuk Kang, Jae Il Shin, Young-Chul Jung, Sun Jae Jung

**Affiliations:** 1 Department of Preventive Medicine, Yonsei University College of Medicine, 50-1 Yonsei-ro, Seodaemun-gu, Seoul, 03722 South Korea; 2 Department of Public Health, Yonsei University Graduate School, Seoul, South Korea; 3 Department of Psychiatry, Yonsei University College of Medicine, Seoul, South Korea; 4 Department of Paediatrics, Yonsei University College of Medicine, Seoul, South Korea

**Keywords:** Anxiety, Depression, Social distance, Mental health, Systemic reviews

## Abstract

**Background:**

Public isolated due to the early quarantine regarding coronavirus disease 2019 (COVID-19) increasingly used more social media platforms. Contradictory claims regarding the effect of social media use on mental health needs to be resolved. The purpose of the study was to summarise the association between the time spent on social media platform during the COVID-19 quarantine and mental health outcomes (i.e., anxiety and depression).

**Methods:**

Studies were screened from the PubMed, Embase, and Cochrane Library databases. Regarding eligibility criteria, studies conducted after the declaration of the pandemic, studies that measured mental health symptoms with validated tools, and studies that presented quantitative results were eligible. The studies after retrieval evaluated the association between time spent on social media platform and mental health outcomes (i.e. anxiety and depression). The pooled estimates of retrieved studies were summarised in odds ratios (ORs). Data analyses included a random-effect model and an assessment of inter-study heterogeneity. Quality assessment was conducted by two independent researchers using the Risk of Bias Assessment Tool for Nonrandomized Studies (RoBANS). This meta-analysis review was registered in PROSPERO (https://www.crd.york.ac.uk/PROSPERO/, registration No CRD42021260223, 15 June 2021).

**Results:**

Fourteen studies were included. The increase in the time spent using social media platforms were associated with anxiety symptoms in overall studies (pooled OR = 1.55, 95% CI: 1.30–1.85), and the heterogeneity between studies was mild (I^2^ = 26.77%). Similarly, the increase in social media use time was also associated with depressive symptoms (pooled OR = 1.43, 95% CI: 1.30–1.85), and the heterogeneity between studies was moderate (I^2^ = 67.16%). For sensitivity analysis, the results of analysis including only the “High quality” studies after quality assessment were similar to those of the overall study with low heterogeneity (anxiety: pooled OR = 1.45, 95% CI: 1.21–1.96, I^2^ = 0.00%; depression: pooled OR = 1.42, 95% CI: 0.69–2.90, I^2^ = 0.00%).

**Conclusions:**

The analysis demonstrated that the excessive time spent on social media platform was associated with a greater likelihood of having symptoms of anxiety and depression.

**Supplementary Information:**

The online version contains supplementary material available at 10.1186/s12889-022-13409-0.

## Introduction

Despite the tremendous worldwide efforts including the introduction of vaccines, developing therapeutics and social distancing, the coronavirus outbreak is not expected to dampen due to the continuous emergence of new viral strains and difficulty in effective quarantine interventions. As a result of strong quarantine measures, private meetings, gatherings, and physical contact with intimate relatives have been reduced [1]. Prolonged social distancing and loss of intimate interpersonal contact increase feelings of frustration, boredom, anxiety, and potentially depression [2].

Studies have found that young, socially active populations or workers at high risk of infection, especially college students and frontline healthcare workers, bear a disproportionate burden of mental health problems worldwide (e.g., high levels of anxiety and depression), highlighting the need for appropriate intervention in these populations [3, 4].

Social media in digital platforms is reportedly considered as a new channel of communication that could relieve aforementioned negative aspects of isolation through helping people escape negative emotions [5], projecting their personality as they desire, and evoking the impression of gaining back some control [6]. Social media may be helpful for relieving anxiety and depression by providing information regarding the pandemic [7, 8].

However, prolonged use of social media by the isolated could be a double-edged sword that can adversely affect mental health due to sustained exposure to excessive information and misinformation [9–11]. While social media in digital platforms does help to promote social inclusion among adolescents and young adults, the risk associated with their excessive or problematic use cannot be overlooked [12]. Due to conflicting evidence and views regarding the effect of social media platform on the mental health, the recommendation for the use of social media in pandemic has been questioned.

Therefore, a meta-analysis was conducted to solve the contradictory effects of social media platform on anxiety and depression based on studies reporting an association between the use of social media and mental health outcomes (i.e., anxiety and depression) on the pandemic setting.

## Methods

### Eligibility criteria

Studies were included which met the following criteria: (1) use of the English language; (2) conducted after March 11, 2020 (date the WHO declared a pandemic) and published by December 20, 2020; (3) collected data using a validated tool of mental health symptoms (e.g., Patient Health Questionnaire: PHQ9, Generalized Anxiety Disorder-7 items: GAD-7); (4) full texts available; (5) measured time spent on social media platform in either continuous or categorical variable; (5) provided their results in OR, β, and/or Pearson’s r, and (6) studies measured mental health symptoms such as anxiety and depression.

Studies with the following characteristics were excluded: (1) Studies examined traditional social media (e.g., television and radio); (2) case reports, letters, comments, and narrative reviews without quantitative results, and (3) studies using a language other than English.

Studies investigating the association between time spent on social media and mental health outcomes (e.g., anxiety and depression) were summarised in Supplementary Material 1. The pooled effect size of this meta-analysis was mainly presented in an odds ratio (Fig. 2).

### Study selection

The search strategy principles were as follows: (1) “Social media” or individual names of social media in the title, keyword and abstract results; (2) Terms referring to mental health with COVID-19 specified in the title (e.g. depression, anxiety or blue).

A systematic literature search of the PubMed, Embase, and Cochrane Library databases was performed to identify studies. Publication date restrictions are from March 2020 to December 20, 2020. The search terms for a systematic search were as following: (1) (“COVID-19“ OR “corona“) AND (“mental health” OR depress* OR anxiety) AND (“social media” OR “Instagram” OR “Facebook” OR “twitter”) for PubMed, (2) (“coronavirus disease 2019’/exp/mj) AND (“mental health“/exp/mj OR “depression“/exp OR “anxiety“/exp) AND (“social media”/exp./mj OR “Facebook”/exp. OR “twitter”/exp. OR “Instagram“/exp) for Embase; (3) (“COVID-19″ OR “corona”) AND (“mental health“ OR depress* OR “anxiety”) AND (“social media“ OR ‘Instagram” OR “Facebook” OR “twitter”) for Cochrane Library.

Articles were first screened by reviewing titles, followed by a full-text review. Every selection stage involved three independent researchers (two medical doctors [SJJ and YRL] and one graduate student from the Epidemiology Department [YJJ]). Every article was independently evaluated by two researchers (YJJ and YRL) in first hand, and a third researcher (SJJ) mediated the final selection in case of differences in opinion.

### Data extraction

Study data were extracted by two independent researchers (YRL and YJJ). A single author first extracted the information and a second author checked for accuracy. The extracted information is as follows: country of study, participant group sampled, age group of sample, date of data collection, mental health measures, effect size information, social media use time, and whether the adjustment was made for each analysis (see Supplementary Material 1). Studies were subdivided into categories according to the summary estimate of effect sizes (odds ratio [OR], beta estimate from multiple linear regression [β], and correlation coefficient [Pearson’s r]).

### Exposure variables

The final studies after retrieval measured the amount of time spent on social media, which was either categorical or continuous variables (see Supplementary Material 1). It was measured based on the response to an item in the questionnaire: “How often were you exposed to social media? [categorical]” and “How long (in hours) were you exposed to social media? [continuous].” The measurement of exposure was expressed in different wordings as follows: “Less” vs. “Frequently,” “Less” vs. “Often”, “less than 1 hour” vs. “2 hours or more,” or “less than 3 hours” vs. “3 hours or more.” To calculate the overall effect, these individually measured exposure levels were operationally redefined (e.g., “Less” and “Few” were considered the same as “less than 2 hours;” “less than 1 hour,” “Frequently,” and “Often” were treated the same as “2 hours or more” and “3 hours or more”).

### Outcome variables

The outcomes of included studies were “anxiety”, and “depression”. Anxiety was ascertained by using GAD-7 (cut-off: 10+), DASS-21, and PHQ-9, while depression was measured using PHQ-9 (cut-off: 10+), WHO-5 (cut-off: 13+), and GHQ-28 (cut-off: 24+). Anxiety and depression measured by using screening tools with cut-offs presented results in odds ratios (see Supplementary Material 1).

### Statistical analysis

All statistical analyses and visualisations were performed with the “meta,” “metaphor,” and “dmeter” package of R version 3.6.3 (https://cran.r-project.org/), using a random-effect model [13–15]. The effect measures were odds ratio, regression coefficient, and Pearson’s r, which calculated the association between the increase in social media use time and anxiety and depressive symptoms. In each study, the association with the mental health level of the social media frequent use group (compared to the low frequency group) was calculated as the odds ratio, and the association with the increase in the mental health level per hour increase was calculated as the regression coefficient (β) and Pearson’s r. Statistics used for calculating pooled effects (e.g., odds ratio, regression coefficient, and Pearson’s r) were utilized as its adjusted value with covariates from each study, not the unadjusted crude values.

The pooled effect sizes, Cochrane’s Q, and I^2^ to assess heterogeneity were calculated. The pooled effect sizes, CIs, and prediction intervals were calculated by estimating the pooled effect and CIs using the Hartung-Knapp-Sidik-Jonkman method, which is known as the one of the most conservative methods [16]. The degree of heterogeneity was categorised as low, moderate, or high with threshold values of 25, 50, and 75%, respectively [17]. Possible causes of heterogeneity among study results were explored by statistical methods such as influential analysis, the Baujat plot, leave-one-out analysis, and Graphic Display of Heterogeneity analysis [18]. In addition, publication bias was assessed using funnel plots, Egger’s tests, and the trim-and-fill method [19].

### Quality assessment

Quality assessment was conducted by two independent researchers, a psychiatrist (SHK) and an epidemiologist (YRL), using the Risk of Bias Assessment Tool for Nonrandomized Studies (RoBANS), which can assess cross-sectional studies [20]. RoBANS has been validated with moderate reliability and good validity. RoBANS applies to cross-sectional studies and comprises six items: participant selection, confounding, exposure measurement, blinding of outcome assessments, missing outcomes, and selective reporting of outcomes. Each item is measured as having a “high risk of bias,” “low risk of bias,” or “uncertain.” For example, based on “participant selection,” each researcher marked an article as having a “high risk of bias” if, for example, the patient definitions of depression were generated by self-reported data. In cross-sectional studies, misclassification cases due to an unreliable self-contained questionnaire for categorizing depressive patients were rated as “high risk.” For the qualitative assessment, studies with two or more “high risk of bias” grades were then classified as “low quality”. The study was rated as “high quality” only if the evaluation of both raters was congruent. For sensitivity analysis, additional analysis including only “high quality” studies was conducted and it compared with the pooled estimates of overall results (see Table 1).

**Table 1:** Association between social media use and anxiety^a^ and depression^b^

	Studies, n (participants)	Pooled		I^**2**^ (%)
		effect sizes [95% CI]	Q-statistics	
**Anxiety symptoms**				
Odds ratio				
Overall studies	**6 (9579)**	**1.55 [1.30–1.85]**	**6.84**	**26.94**
Quality assessment				
High Quality	**4 (7599)**	**1.45 [1.21–1.73]**	1.76	0.00
β ^c^	3 (2376)	0.05 [−0.32–0.41]	8.36	76.07
Pearson’s r ^d^	**4 (2483)**	**0.18 [0.10–0.27]**	11.13	73.04
**Depressive symptoms**				
Odds ratio				
Overall studies	**6 (13241)**	**1.43 [1.14–1.80]**	15.22	67.16
Quality assessment				
High Quality	2 (4481)	1.42 [0.69–2.90]	0.33	0.00
β ^c^	3 (2574)	0.08 [0.01–0.14]	0.82	0.00

^a^ Anxiety symptoms were ascertained using the GAD-7 (cut-off: 10), GHQ-28, GAD-2, PHQ-4, GAD-2, SAS, and DASS-21

^b^ Depressive symptoms were ascertained by the DASS-21, WHO-5 (cut-off: 13), PHQ-9 (cut-off:10), GHQ-28, and PHQ-2

^c^ Beta value calculated by linear regression analysis indicates the change in score over time in hours

^d^ Correlation analysis

*Β* Beta value; *CI* Confidence Interval; *DASS* Subscale scores of Depression, Anxiety, and Stress Scale; *GAD* Generalized Anxiety Disorder; *GHQ-28* General Health Questionnaire-28; *PHQ* Patient Health Questionnaire; *SAS* Self-Rating Anxiety Scale

* Significant results are in bold text

### Ethical approval

The preferred reporting items for systematic reviews and meta-analyses (PRISMA) guidelines 2020 were followed for this study. No ethical approval and patient consent are required since this study data is based on published literature. This meta-analysis review was registered with PROSPERO (https://www.crd.york.ac.uk/PROSPERO/, registration No CRD42021260223, 15 June 2021).

## Results

### Included and excluded studies

**Fig. 1:**
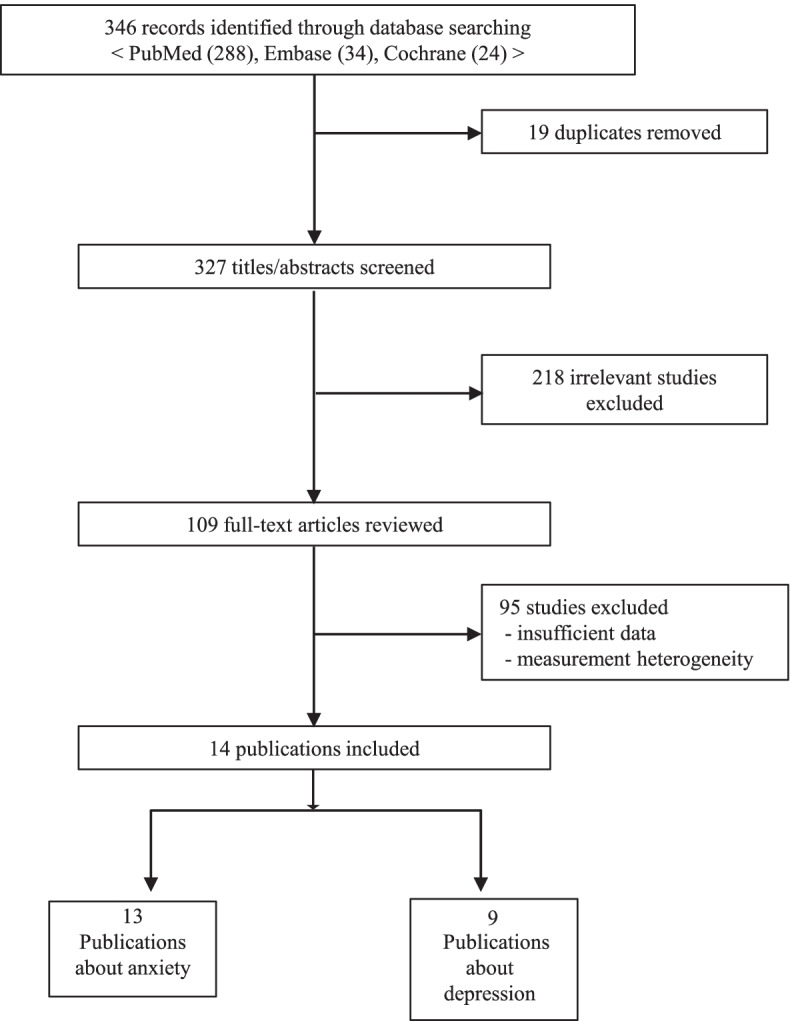
Flowchart of literature search and selection of the publications

Total of 346 studies were selected from the database search (288 from PubMed, 34 from Embase, and 24 from the Cochrane Library). After removing 19 duplicate publications, 327 studies were included for the title and full-text review (see Fig. 1). Non-original studies and those conducted with irrelevant subjects (*n* = 218) were excluded. Another 95 studies were excluded finally due to inconsistent study estimates. As summarised in Supplementary material 1 and 8, 13 papers studied anxiety as an outcome (6 studies in odds ratio, 3 in regression coefficient, 4 in Pearson’s r), and a total of 9 papers studied depression as an outcome (6 studies in odds ratio, 3 in regression coefficient). Each of the final distinct 14 studies (after excluding duplicate studies) measured multiple mental health outcome variables (i.e., anxiety and depression), and pooled effect sizes were calculated for each outcome. Six studies that dealt with anxiety symptoms and six with depression (Supplementary Material 1–1-1, 1–2-1) reported ORs and their 95% confidence intervals (CIs) (*n* = 9579 and *n* = 13,241 for anxiety and depressive symptoms, respectively). Three studies each on anxiety and depression (Supplementary Material 1–1-2, 1–2-2) reported their findings in β (*n* = 2376 and *n* = 2574 for anxiety and depression, respectively). All included studies were cross-sectional studies. The pooled effect size was presented in odds ratio.

### Time spent on social media and mental health outcomes

**Fig. 2:**
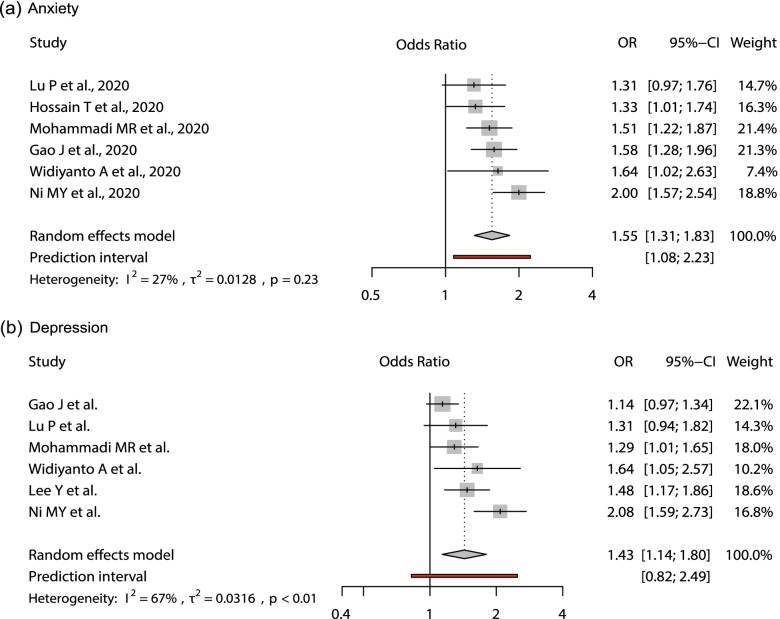
Forest plot for social media exposure and symptoms of mental health (i.e. anxiety & depression) in cross-sectional studies. Estimates presented in odds ratios (OR)

Table 1 shows the result of the meta-analysis about the relationship between time spent on social media and mental health outcomes (i.e., anxiety and depression) of the selected cross-sectional studies. The increase in the time spent using social media platforms were associated with anxiety symptoms in overall studies (pooled OR = 1.55, 95% CI: 1.30–1.85, prediction intervals: [1.08–2.23]), and the heterogeneity between studies was mild (I^2^ = 26.77%) (see Fig. 2). The three cross-sectional studies (presented in β) were insignificant (β = 0.05, 95% CI: − 0.32–0.15; a unit increment of each screening tool score per hour) with relatively high inter-study heterogeneity (I^2^ = 76.07%). The overall estimate of the four cross-sectional studies (Pearson’s r) was 0.18 (95% CI: 0.10–0.27) with high inter-study heterogeneity (I^2^ = 73.04%). The increase in social media use time was also associated with depressive symptoms (pooled OR = 1.43, 95% CI: 1.30–1.85, prediction intervals: [0.82–2.49]), and the heterogeneity between studies was moderate (I^2^ = 67.16%) (see Fig. 2).

### Quality assessment

As result of quality assessment analysis, pooled effect size of studies classified as “high quality” was presented in Table 1. The results were similar to the overall outcome (anxiety: OR = 1.45, 95% CI: 1.21–1.96; depression: OR = 1.42, 95% CI: 0.69–2.90). High-quality studies had low inter-study heterogeneity (anxiety: I^2^ = 0.00%; depression: I^2^ = 0.00%). The kappa statistic (inter-rater agreement) was 33.3%, indicating fair agreement.

### Publication bias

Publication bias was assessed by funnel plot analysis and Egger’s test (Supplementary Material 4–1). Funnel-plot analyses revealed symmetrical results (Supplementary Material 4–2). In addition, all results of the Egger test were statistically insignificant, indicating improbable publication bias. After applying the trim-and-fill method, the funnel plot revealed no asymmetry (Supplementary Material 5), indicating no significant publication bias.

## Discussion

The study aimed to present a comprehensive direction of relevance by analysing studies investigating the association between time spent on social media during the COVID-19 pandemic and mental health symptoms (i.e., anxiety and depressive) among the public. The increase in the time spent on social media in digital platforms was associated with symptoms of anxiety and depression.

The pooled results are in line with previous systematic reviews and meta-analysis performed before the pandemic. A systematic literature review before the COVID-19 outbreak (2019) found that the time spent by adolescents on social media was associated with depression, anxiety, and psychological distress [21]. A meta-analysis of 11 studies (2017) also reported a weak association between social media use and depressive symptoms in children [22]. A meta-analysis of 23 studies (2018) reported significant correlation between social media use and psychological distress [23]. Likewise, this study also observed a similar trend of a negative effect of social media on mental health outcomes in the COVID-19 pandemic. However, the estimates of inter-study heterogeneity of these meta-analysis were relatively high (meta-analysis of 11 studies: I^2^ = 92.4%; meta-analysis of 23 studies: I^2^ = 62.00% for anxiety, I^2^ = 80.58% for depression) compared to the analysis, which implies relatively higher homogeneity of the study population and reliable results.

Unverified information and opinions can be easily disseminated on social media platform and perceived as facts without verification. There has been a stream of news regarding the pandemic, creating a sense of urgency and anxiety. Repeated exposure to the news may affect the construct of external reality and may lead to a delusion-like experience, which has been linked to anxiety and social media overuse [24, 25].

Additionally, discrimination and stigma related to COVID-19 on social media can make people fearful of being infected and exacerbate depression and anxiety [26]. Fear of COVID-19 may be compounded by coexisting depression and anxiety disorders [27]. Due to the high accessibility of social media platform and the ease of socialisation in a controlled setting, individuals with underlying depression may be more drawn to social media interactions rather than face-to-face ones, more so in the pandemic era [28].

Also, implementation of social distancing mandates new norms limiting physical conducts in almost all sectors of life, including educational institutes and vocational venue. Rapid transition to the new remote educational environment and telecommuting may trigger mental health issues [29].

In interpreting the findings of this study, several limitations should be considered. First, all the studies included were cross-sectional design. The possibility of a reverse causal relationship cannot be ruled out. Further studies with longitudinal data are warranted. Second, the results do not represent the general population since most of the studies recruited participants through a web-based survey, which may have had a selection bias. Lastly, some of the analysis showed a relatively high inter-study heterogeneity (range: I^2^ = 0.00–80.53%). The results of the statistical approaches to identify the cause of heterogeneity (i.e. influential analysis, Baujat plot, leave-one-out analysis, and GOSH analysis) were summarised in Supplementary Material 6 and 7.

Despite these limitations, this study exhibits a number of strengths; to the best of our knowledge, the study is the first meta-analysis to examine the relationship between use of social media and mental health outcomes during the COVID-19 pandemic, to validate the results by various verification methods such as trim-and-fill methods, influential analysis, and heterogeneity analysis. In addition, sensitivity analysis was also conducted with unbiased “high quality” studies through quality assessment.

The analysis demonstrates that excessive time spent on social media platform is associated with increased anxiety and depressive symptoms in the pandemic. While social media may be considered as an alternative channel for people to connect with their peers in the pandemic, the findings suggest that excessive use of social media can be detrimental for mental health. Further observation studies with longitudinal design to determine the true effect of social media platform are required.

## Supplementary Information

**Additional file 1.**

**Additional file 2.**

## Data Availability

The data that support the findings of this study are available from the corresponding author upon reasonable request.

## Abbreviations

COVID-19: Coronavirus disease 2019
OR: Odds ratio
CI: Confidence interval
PHQ-9: Patient Health Questionnaire-9
PHQ-2: Shortened version of PHQ
GHQ-28: General Health Questionnaire-28
DASS-21: Depression Anxiety and Stress Scales
WHO-5: World Health Organization-Five Well-Being Index
GAD-7: Generalized Anxiety Disorder-7
GAD-2: Shortened version of GAD
RoBANS: Risk of Bias Assessment Tool for Nonrandomized Studies
