# Supplementary material for: Social media use and mental health during the COVID-19 pandemic in young adults: a meta-analysis of 14 cross-sectional studies
Source: BMC Public Health. 2022 May 17;22:995. doi: 10.1186/s12889-022-13409-0 (PMC9112239; doi:10.1186/s12889-022-13409-0)
Supplement: Supplementary file 1 — Additional file 1. [file 12889_2022_13409_MOESM1_ESM.pdf]

< Supplementary material 1-1-1, Odds ratio >

study_n	author	year	st_design	surv_type	country	total	case	age	sex	exposure	exposure criteria	outcome	outcome	adjustment	or	ci.lb	ci.ub
1	Gao J et al.	2020	4	Online survey among Chinese citizens aged≥ 18 years	Chinese citizens aged≥18 years (from 31 provinces and autonomous regions)	4872		Mean age, Mean (SD), 32.3 (10.0) Range: 18-85	Male: 1,560 (32.3) Female: 3,267 (67.7)	Social media exposure (SME)	During the past week, how often do you were exposed to news and information about COVID-19 on social media/ Less vs. (1) Sometimes, (2) Frequently	Anxiety	GAD-7 (cut-off: 10)	gender, age, marital status, educational level,income	1.72	1.31	2.26
2	Lu P et al.	2020	4	cross-sectional online study among citizens in Wuhan (via WeChat, QQ, and other social media networks)	China (remotely among HWs and general public in Wuhan)	1417	83 (21.7)	Age group, N(%) 18-25: 138 (9.7), 26-35: 441 (31.1), 31-40: 552 (39.0), >40: 286 (20.2)	Male: 235 (16.6) Female: 1,182 (83.4)	Social media exposure (SME)	Few(reference), (1) sometime, (2) often in both healthcare workers and general public	Anxiety	GAD-7 (cut-off: 10)	gender, age, marital status, educational level,income	1.91	1.26	2.9
3	Widiyanto A et al.	2020	4	online survey	China	430	Depression: No, 199 (46.28%); Yes, 231 (53.72%) Anxiety: No, 230 (53.49%);	< 20 190 (44.19) ≥20 240 (55.81)	Male 108 (25.12) Female 322 (74.88)	Social media exposure	How often respondents during 3 months were exposed to news and information about COVID-19 on social media, such as seperti Televisi, Web, Whatsapp, Instagram and etc. (Low(ref), High)	Anxiety	GAD7	gender, age, income, red area zone category (the existence of patients confirmed covid), implementation of Large-scale Social Restriction.	1.64	1.18	3.04
4	Hossain T et al.	2020	4	E-questionnaire, based on the widely used google form, was forwarded to the participants with valid Facebook account	Bangladeshi	880	432(49.1%)	Age group, N(%) ≤20: 150 (17.0), 21-25: 370 (42.0), 26-30: 176 (20.0), >30: 184 (20.9)	Male: 616 (70.0) Female: 264 (30.0)	Social media exposure (SME)	(2) Time spent on SME : ≤2 (ref), 4> hours	Anxiety	GAD-7 (cut-off: 10)	Age, education, marital status, occupation, place of residence, electronic media exposure (EME), types of EME, time spent on EME, self reported health	1.52	1.01	2.31
5	Ni MY et al.	2020	4	online survey via WeChat	China	Total: 1791 community-based adults: 1577 HCW: 214	community-based adults: probable anxiety n=376/probable depression n=303 health professionals: n=47/n=41	18-34 community-based adults: 38.6%, HCW: 58.9% 35-44 28.4% 33.6% >45 33.0% 7.5%	Men community-based adults: 39.2%, HCW: 31.2% Women 60.8% 68.8%	Time spent on COVID-19 news on social media per day	ref) Less than 1 hour (2) 2 hrs or more	Anxiety	GAD-2	gender, marital status, educational level, employment, income	2.78	2.01	3.84
6	Su J et al.	2020	4	online survey via WeChat	China	403	152	mean(SD) 42 (11.2)		Time spent on collecting information social media use	"Time spent on collecting information (ref) <1 hrs (2) 1-2 hrs	anxiety	GAD-7	age, gender, marital status, education level and family income	1.28	0.81	2.01

< Supplementary material 1-1-2, Beta >

study_n	author	year	st_design	surv_type	country	survey period	total	age	sex	exposure	exposure criteria	outcome	outcome criteria	adjustment	b	se	std	others
1	Liu JCJ et al.	2020	4	Online survey (Participants responded to advertisements placed in Facebook/WhatsApp/community groups) aged at least 21 years.	Singapore adult population	March 7–April 21, 2020	1,145	Age group N(%), 21-34 467 (40.8) 35-49 394 (34.4) 50-64 220 (19.2) ≥65 64 (5.6)	Male: 382 (33.4) Female: 715 (62.4) Did not answer: 48 (4.2)	the amount of time spent receiving and discussing COVID-19 news	(unit: hours/day)	Anxiety	Subscale scores of Depression, Anxiety, and Stress Scale (DASS-21)	time spent getting COVID-19 updates, number of COVID-19 rumors heard, number of COVID-19 rumors shared, number of COVID-19 rumors believed, use of government's WhatsApp channel, trust in government's WhatsApp messages, likelihood of sharing government's WhatsApp messages, lockdown, local COVID-19 cases to date, age, gender, ethnicity, religion, marital status, educational level, house type, household	0.002	0.09	0	
2	Chao M et al.	2020	4	internet based survey using Tencent questionnaire, through weChat	China	2020-01-28	917	Mean(SD): 24.59(9.5)		media use	sum of hours spent	Anxiety	DASS-21	same as above	0.141		1	
3	Ruggieri S et al.	2020	2	online survey through a social media platform	Italian	between the 7th of March and 14th of April 2020	113	mean(SD) 32.05 (8.01) at T1 32.13 (7.65) at T2 32.35 (7.87) at T3	45 men and 68 women at T1 24 men and 51 women at T2 27 men and 53 women at T3	average daily time spent on SNS	TIme spent on social media	anxiety	DASS-21		0.3			SEM

< Supplementary material 1-1-3, (r) >

study_n	author	year	st_design	surv_type	country	survey period	total	age	sex	exposure	exposure criteria	outcome	outcome criteria	r	p
1	Chao M et al.	2020	4	internet based survey using Tencent questionnaire, through weChat	China	43858	917	Mean(SD): 24.59(9.5)		media use, media engagement	sum of hours spent	Anxiety	DASS-21	0.091	p < 0.01
2	Kazan Kızılkurt O et al.	2020	4	Online study, tool: SurveyMonkey (https://tr.surveymonkey.com/), snowball sampling technique	Turkey	March 28–April 04, 2020	1046	Mean age(SD), 37.1 (12.8), Range: 18−65	Male: 690 (66.0), Female: 356 (34.0)	Time spent on social media during 2 weeks	"What is the time you spent on social media during the past 2 weeks?" (unit: hour)	Anxiety	HAS, 18 -item self -report index, 3-point Likert scale (0−3), Range: 0−54	0.19	N/A (p<0.01)
3	Drouin M et al.	2020	4	online survey via social media announcements and sharing via e-mail listervs	U.S. residents at least 18 years of age	March 20–25, 2020	260	Parents, Mean (SD): 40.07 (7.51); Range=22−62	Male: 31 (11.9) Female: 220 (84.6) Did not respond: 9 (3.5)	Social media/social tech to connect	(1) Parent: Active social media use; 4-point Likert scale; 1 (No)-4 (Yes, a lot) (2) Child: social media/social tech to connect; 5-point Likert scale; 0 (Does not use technology)-4(Yes, a lot)	Parent anxiety	2 items from Patient Health Questionnaire-4(PHQ-4): GAD-2, 4-point Likert scale ranging from 0 ("not at all") to 3 ("nearly every day"), Range: 0-6	0.17	p<0.01
4	Drouin M et al.	2020	4	online survey via social media announcements and sharing via e-mail listervs	U.S. residents at least 18 years of age	March 20–25, 2020	260	Parents, Mean (SD): 40.07 (7.51); Range=22−62	Male: 31 (11.9) Female: 220 (84.6) Did not respond: 9 (3.5)	Social media/social tech to connect	(1) Parent: Active social media use; 4-point Likert scale; 1 (No)-4 (Yes, a lot) (2) Child: social media/social tech to connect; 5-point Likert scale; 0 (Does not use technology)-4(Yes, a lot)	Child anxiety	1 items from Patient Health Questionnaire-4(PHQ-4): 4-point Likert scale ranging from 0 ("not at all") to 3 ("nealy every day"), Range: 0-3("Over the last 2 weeks, how often has YOUR CHILD(REN) felt nervous, anxious, or on edge?")	0.3	p<0.001

< Supplementary material 1-2-1, Odds ratio >

study_n	author	year	st_design	surv_type	country	total	case	age	sex	exposure	exposure criteria	outcome	outcome	adjustment	or	ci.lb	ci.ub
1	Gao J et al.	2020	4	Online survey among Chinese citizens aged≥18 years	Chinese citizens aged≥18 years (from 31 provinces and autonomous regions)	4872		Mean age, Mean (SD) 32.3 (10.0) Range: 18–85	Male: 1,560 (32.3) Female: 3,267 (67.7)	Social media exposure (SME)	During the past week, how often do you were exposed to news and information about COVID-19 on social media (such as Sina weibo, Zhihu, Douban, WeChat and etc.) – 5-point Likert scale (1. Never –5. Very often)/ Less vs. (1) Sometimes, (2) Frequently	Depression	The Chinese version of WHO-Five Well-Being Index (WHO-5) (cut-off: 13)	gender, age (10-year categories), educational level (junior high school, senior high school, college and master and higher), marital status (recoded into married and other [including unmarried, divorced, and widowed]), self-rated health (categorized as excellent, very good and good or low), occupation (students/retired, health care worker and others), cities (Wuhan and others), area (urban and rural)	1.18	0.96	1.45
2	Lu P et al.	2020	4	cross-sectional online study among citizens in Wuhan (via WeChat, QQ, and other social media networks)	China (remotely among HWs and general public in Wuhan)	1417	83 (21.7)	Age group, N (%) 18-25: 138 (9.7), 26–35: 441 (31.1), 31–40: 552 (39.0), >40: 286 (20.2)	Male: 235 (16.6) Female: 1,182 (83.4)	Social media exposure (SME)	Few (reference), (1) sometime, (2) often in both healthcare workers and general public	Depression	PHQ-9 (cut-off:10)	gender, age, marital status, educational level, income	1.64	1.04	2.6
4	Mohammadi MR et al.	2020	4	online questionnaire	Iranian citizens	1881	713	<20 42 (2.23) 21-30 390 (20.73) 31-40 758 (40.3) 41-50 422 (22.43) 51-60 210 (11.16) >61 59 (3.14)	Male 651 (34.61) Female 1,230 (65.39)	Media Consumption	(1) during the past 2 weeks, to what extent did you obtain news and information about COVID-19 from national media?	Depression	GHQ-28	demographic variables, public trust, media related variables, and prior anxiety to illness	1.29	1.007	1.653
5	Widiyanto A et al.	2020	4	online survey	China	430	Depression: No, 199 (46.28%); Yes, 231 (53.72%) Anxiety: No, 230 (53.49%); Yes, 200 (46.51%)	< 20 190 (44.19) ≥20 240 (55.81)	Male 108 (25.12) Female 322 (74.88)	Social media exposure	How often respondents during 3 months were exposed to news and information about COVID-19 on social media, such as seperti Televisi, Web, Whatsapp, Instagram and etc.	Depression	WHO-5	gender, age, income, red area zone category (the existence of patients confirmed covid), implementation of Large-scale Social Restriction.	1.64.	1.05	2.57
6	Lee Y et al.	2020	4	Telephone/Wechat survey, national convenience sample of 2574 health -care workers and 490 non-medical workers	China	3064	N/A	N/A	N/A	Social media use for COVID-19 information	<3 (ref), ≥3 hours per day	Depression	PHQ-9 (cut-off: (1) 5, (2) 10)	Not mentioned	1.475	1.167	1.865
7	Ni MY et al.	2020	4	online survey via WeChat	China	Total: 1791 community-based adults: 1577 HCW: 214	community-based adults: probable anxiety n=376/probable depression n=303 health professionals: n=47/ n=41	18-34 community-based adults: 38.6%, HCW: 58.9% 35-44 28.4% 33.6% >45 33.0% 7.5%	Men community-based adults: 39.2%, HCW: 31.2% Women 60.8% 68.8%	Time spent on COVID-19 news on social media per day	ref) Less than 1 hour (1) 1-2 hrs (2) 2 hrs or more	Depression	PHQ-2	gender, marital status, educational level, employment, income	3.08	2.16	4.4

study_n	author	year	st_design	surv_type	country	total	exposure	exposure criteria	outcome	outcome criteria	b	se	std
1	Liu JC et al.	2020	4	Online survey (Participants responded to advertisements placed in Facebook/WhatsApp/community groups) aged atleast 21 years.	Singapore adult population	1145	the amount of time spent receiving and discussing COVID-19 news	(unit: hours/day)	Depression	Subscale scores of Depression, Anxiety, and Stress Scale (DASS-21)	0.055	0.103	0
2	Chao M et al.	2020	4	internet based survey using Tencent questionnaire, through weChat	China	917	media use	sum of hours spent	Depression	DASS-21	0.088 N/A	1	
3	Ruggieri S et al.	2020	2	online survey through a social media platform	Italian	113	average daily time spent on SNS	TIme spent on social media	Depression	DASS-21	0.35 N/A	1	

< Supplementary material 1-3-1, excluded studies, anxiety, Odds ratio >

study_n	selec_no	no	author	year	st_design	surv_type	country	total	case	age	sex	exposure	exposure criteria	outcome	outcome	adjustment	or	ci.lb	ci.ub	excluded
3	60	PM111	Mohammadi MR et al.	2020	4	online questionnaire	Iranian citizens	1881	713	<20 42 (2.23)<br>21-30 390 (20.73)<br>31-40 758 (40.3)<br>41-50 422 (22.43)<br>51-60 210 (11.16)<br>>61 59 (3.14)	Male 651 (34.61)<br>Female 1,230 (65.39)	Media Consumption	(1) during the past 2 weeks, to what extent did you obtain news and information about COVID-19 from national media? Likert 5 point scale "0-never" to "4-very much"	anxiety	GHQ-28	demographic variables, public trust, media related variables, and prior anxiety to illness	1.513	1.222	1.872	Heterogeneous exposure variables

< Supplementary material 1-3-2, excluded studies, anxiety, beta >

study_n	selec_no	no	author	year	st_design	surv_type	country	survey period	total	case	age	sex	exposure	exposure criteria	outcome	outcome criteria	adjustment	b	se	std	others	excluded
1	23	PM219	Drouin M et al.	2020	4	online survey via social media announcements and sharing via e-mail listervs	U.S. residents at least 18 years of age	March 20-25, 2020	260		Parents, Mean (SD): 40.07 (7.51); Range=22-62	Male: 31 (11.9) Female: 220 (84.6) Did not respond: 9 (3.5)	Social media/social tech to connect	**(1) Parent:** Active social media use; 4-point Likert scale; 1 (No)-4 (Yes, a lot) **(2) Child:** social media/social tech to connect; 5-point Likert scale; 0 (Does not use technology)-4 (Yes, a lot)	Parent anxiety	**2 items from Patient Health Questionnaire-4 (PHQ-4): GAD-2**, 4-point Likert scale ranging from 0 ("not at all") to 3 ("nearly every day"), Range: 0-6	None	0.17	N/A (p<0.01)	1	SEM	Heterogeneous exposure measurement
2	23	PM219	Drouin M et al.	2020	4	online survey via social media announcements and sharing via e-mail listervs	U.S. residents at least 18 years of age	March 20-25, 2020	260		Parents, Mean (SD): 40.07 (7.51); Range=22-62	Male: 31 (11.9) Female: 220 (84.6) Did not respond: 9 (3.5)	Social media/social tech to connect	**(1) Parent:** Active social media use; 4-point Likert scale; 1 (No)-4 (Yes, a lot) **(2) Child:** social media/social tech to connect; 5-point Likert scale; 0 (Does not use technology)-4 (Yes, a lot)	Child anxiety	**1 items from Patient Health Questionnaire-4 (PHQ-4):** 4-point Likert scale ranging from 0 ("not at all") to 3 ("nearly every day"), Range: 0-3 ("Over the last 2 weeks, how often has YOUR CHILD (REN) felt nervous, anxious, or on edge?")	None	0.11	N/A (p<0.05)	1	SEM	Heterogeneous exposure measurement
3	90	PM048	Soroya SH et al.	2020	4	web-based questionnaire in Webropol	Finland	from April 4th to April 29th	321		<20 29 (9%)<br>21–25 133 (41%)<br>26–34 68 (21%)<br>35–44 43 (13%)<br>45–54 29 (9%)<br>>55 19 (6%)	Male 133 (41%) Female 179 (56%)	Social Media	Frequency/<br>6-point scale [1 = never, 2 = less than one hour, 3 = 1–2 h, 4 = 3–4 h, 5 = 5–6 h, 6 = more than 6 h]	information anxiety	5-point Likert scale from 1 ("strongly disagree") to 5 ("strongly agree")	None	0.114	0.053		SEM	Heterogeneous exposure measurement
4	36	PM164	Kazan Kızılkurt O et al.	2020	4	Online study, tool: SurveyMonkey (https://tr.surveymonkey.com/), snowball sampling technique	Turkey	March 28-April 04, 2020	1,046	Psychiatric treatment history (25%)	Mean age (SD), 37.1 (12.8), Range: 18-65	Male: 690 (66.0), Female: 356 (34.0)	Time spent on social media during 2 weeks	"What is the time you spent on social media during the past 2 weeks?" (Less than 1 h<br>1–3 h<br>4–6 h<br>More than 7 h)	Anxiety	HAS, 18-item self-report index, 3-point Likert scale (0-3), Range: 0-54	Chronic medical disease, psychiatric treatment history, perception of self, BHS, duration following coronavirus related news	0.01	N/A (p=0.002)	1		Heterogeneous exposure measurement
5	46	PM138	Liu C et al.	2020	4	Online survey by a data-collection service provider (i.e., Changsha Ranxing IT Ltd.)	China (30 provinces in mainland China)	The first two weeks of April 2020	1,118		Age group (%),<br>Below 18 4.1%<br>18–25 30.6%<br>26–30 22.6%<br>31–35 23.5%<br>36–40 9%<br>41–50 7.6%<br>Above 50 2.5%	Male: 45.9% Female: 54.1%	Media use during the pandemic of COVID-19 (from late January to February)	Time spent on coronavirus information each day, 5-point Likert scale (1, hardly ever-5, more than 5 hours/day)	Anxiety (outcome)	3-items 5-point Likert scale; adapted from Self-Rating Anxiety Scale (SAS), Range: 3-15	Time Spent on COVID-19 Information controlled, **media vicarious traumazation**	-0.02	N/A (p>0.05)	0	Mediation	Heterogeneous exposure measurement
9	105	PM007	Zhao N et al.	2020	4	online survey	China	24 March to 1 April 2020	512		Mean(SD) 22.12 (2.47)	male 192 (37.5%) female 320 (62.5%)	social media use	summing up the total daily hours	Anxiety	GAD-7	traditional and internet media usage	0.12		1	CI or t not provided	Heterogeneous exposure measurement

< Supplementary material 1-3-3, excluded studies, depression, beta >

study_n	selec_no	no	author	year	st_design	surv_type	country	survey period	total	age	sex	exposure	exposure criteria	outcome	outcome criteria	adjustment	b	std	others	excluded
4	105	PM007	Zhao N et al.	2020	4	online survey	China	24 March to 1 April 2020	512	Mean(SD) 22.12 (2.47)	male 192 (37.5%) female 320 (62.5%)	social media use	summing up the total daily hours	Depression	PHQ-9	same as above	0.11	1	CI or t not provided	Heterogeneous exposure measurement

< Supplementary material 1-3-4, excluded studies, depression, Odds ratio >

study_n	selec_no	no	author	year	st_design	surv_type	country	survey period	total	case	age	sex	exposure	exposure criteria	outcome	outcome	adjustment	or	ci.lb	ci.ub	excluded
4	60	PM111	Mohammadi MR et al.	2020	4	online questionnaire	Iranian citizens	between March 5, 2020 to March 22, 2020	1881	713	<20 42 (2.23)<br>21-30 390 (20.73)<br>31-40 758 (40.3)<br>41-50 422 (22.43)<br>51-60 210 (11.16)<br>>61 59 (3.14)	Male 651 (34.61)<br>Female 1,230 (65.39)	Media Consumption	(1) during the past 2 weeks, to what extent did you obtain news and information about COVID-19 from national media?	Depression	GHQ-28	demographic variables, public trust, media related variables, and prior anxiety to illness	1.29	1.007	1.653	No description of exposure variable measurement

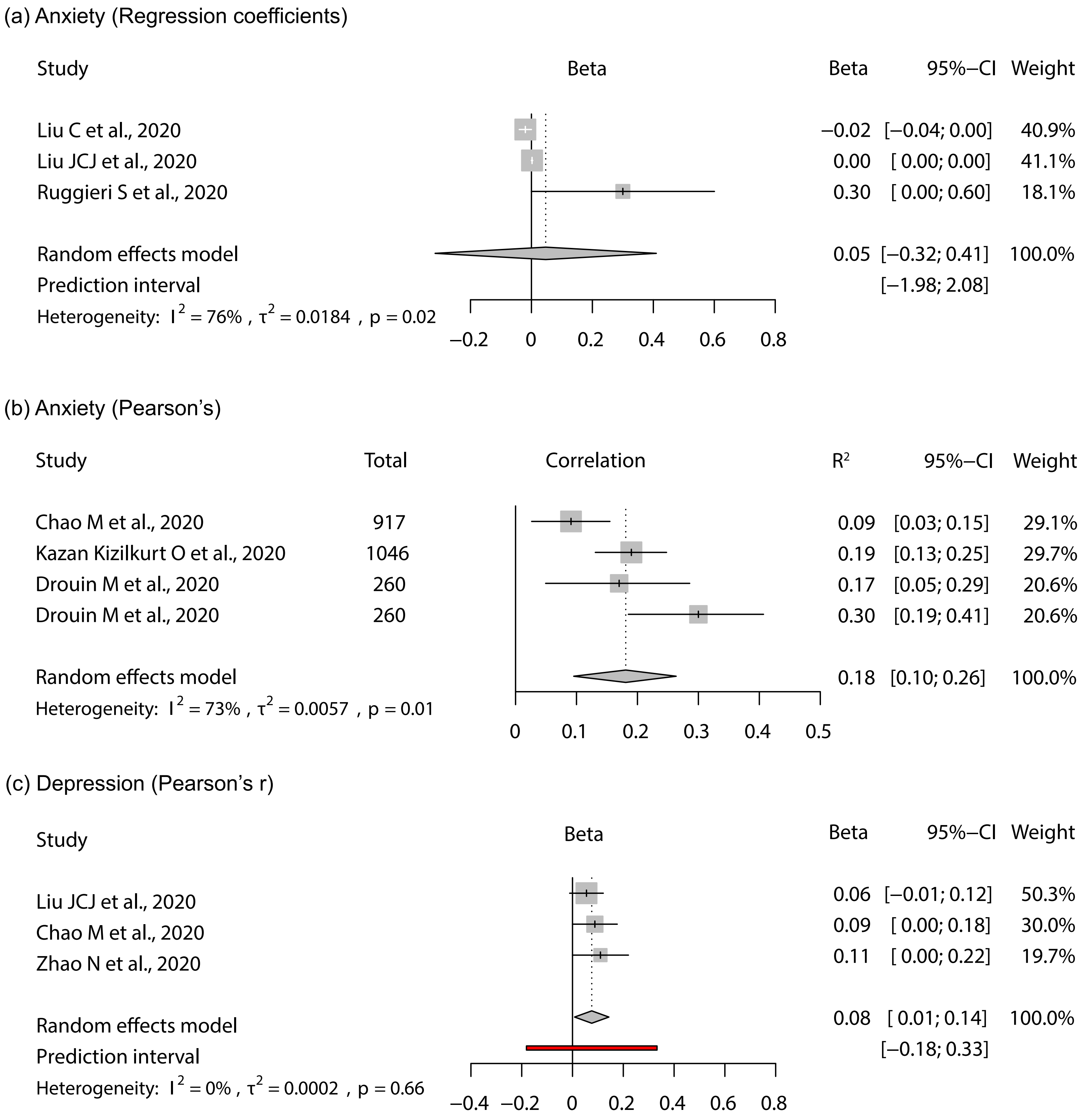

Supplementary material 2. Forest plots for social media exposure and risk of mental health outcomes (i.e. anxiety and depression) in cross-sectional studies.

<Supplemenatry Material 3-1. Risk of Bias of cohort studies by RoBANS>

	study, year	1. selection of participants	2. Confounding	3. Measurement of exposure	4. Blinding of outcome assessments	5. Incomplete outcome data	6. Selective outcome reporting	overall	weight
OR_Anxiety									
	Widiyanto A et al., 2020	Low	Low	High	Low	Unclear	Low	Low	6.3
	Su J et al., 2020	Low	Low	High	Low	Low	Low	Low	10.1
	Ni MY et al., 2020	Low	Unclear	High	Low	Unclear	Low	High	17
	Lu P et al., 2020	Low	Low	High	Low	Unclear	Low	Low	13
	Hossain T et al., 202	Low	Low	High	Low	Low	Unclear	Low	14.5
	Gao J et al., 2020	Low	Low	High	Low	Low	Low	Low	19.5
OR_Depression									
	Widiyanto A et al., 2020	High	Low	High	Low	Unclear	Low	High	11.6
	Ni MY et al., 2020	Low	Unclear	High	Low	Unclear	Low	Low	14.6
	Mohammadi MR et al., 2020	Low	Low	High	Low	Unclear	Low	Low	14.9
	Lu P et al., 2020	Low	Low	High	Low	Unclear	Low	Low	13.6
	Lee Y et al., 2020	Low	Low	High	Low	Unclear	Low	Low	15.1
	Gao J et al., 2020	High	Low	High	Low	Low	Low	Low	16.1
B_Anxiety									
	Chao M et al., 2020	High	Unclear	High	Low	Unclear	Low	Low	8.5
	Ruggieri S et al., 2020	Unclear	Low	High	Low	Low	Low	Low	3.4
	Liu JCJ et al., 202	High	Low	High	Low	Low	Low	Low	15.2
B_Depression									
	Chao M et al., 2020	Low	Unclear	High	Low	Unclear	Low	High	31
	Ruggieri S et al., 2020	Unclear	Low	High	Low	Low	Low	Low	27.5
	Liu JCJ et al., 2020	High	Low	High	Low	Low	Low	High	34.5
B_Stress									
	Chao M. et al., 2020	Unclear	Unclear	High	Low	Unclear	Low	High	10.6
	Ruggieri S et al., 2020	Low	Low	High	Low	Low	Low	Low	7.9
	Liu JCJ et al., 202	Low	Low	High	Low	Low	Low	Low	11
R_Anxiety									
	Chao M et al., 2020	Unclear	Unclear	High	Low	Unclear	Low	High	29.1
	Kazan Kızılkurt O et al., 2020	Unclear	High	High	Low	Unclear	Low	High	29.7
	Drouin M et al., 2020	Low	Low	High	Low	Low	Low	Low	20.6
	Drouin M et al., 2020	Low	Low	High	Low	Low	Low	Low	20.6
R_Stress									
	Chao M et al., 2020	High	Unclear	High	Low	Unclear	Low	High	34.2
	Hong W et al., 2020	Low	High	High	Low	Low	Low	Low	26.1
	Drouin M et al., 2020	Low	Low	High	Low	Low	Low	Low	19.8
	Drouin M et al., 2020	Low	Low	High	Low	Low	Low	Low	19.8

<Supplemenatry Material 3-2. Risk of Bias of cohort studies by RoBANS>

	study, year	1. selection of participants	2. Confounding	3. Measurement of exposure	4. Blinding of outcome assessments	5. Incomplete outcome data	6. Selective outcome reporting	overall	weight
OR_Anxiety									
	Widiyanto A et al., 2020	Low	Low	High	Low	Uncertain	Low	Low	6.3
	Su J et al., 2020	Low	High	High	Low	High	Low	High	10.1
	Ni MY et al., 2020	Low	High	High	Low	Uncertain	Low	High	17
	Lu P et al., 2020	Low	Low	High	Low	Low	Low	Low	13
	Hossain T et al., 202	Low	Low	High	Low	Low	Low	Low	14.5
	Gao J et al., 2020	Low	Low	High	Low	Uncertain	Low	Low	19.5
OR_Depression									
	Widiyanto A et al., 2020	High	Low	High	Low	Uncertain	Low	High	11.6
	Ni MY et al., 2020	Low	High	High	Low	Uncertain	Low	High	14.6
	Mohammadi MR et al., 2020	High	Low	High	Low	High	Low	High	14.9
	Lu P et al., 2020	Low	Low	High	Low	Low	Low	Low	13.6
	Lee Y et al., 2020	Low	Uncertain	High	Low	Uncertain	Low	Low	15.1
	Gao J et al., 2020	High	Low	High	Low	Uncertain	Low	High	16.1
B_Anxiety									
	Chao M et al., 2020	Low	Low	Low	Low	Uncertain	Low	Low	8.5
	Ruggieri S et al., 2020	Low	High	High	Low	High	Low	High	3.4
	Liu JCJ et al., 202	Low	Low	Low	Low	High	Low	Low	15.2
B_Depression									
	Chao M et al., 2020	Low	Low	Low	Low	Uncertain	Low	Low	31
	Ruggieri S et al., 2020	Low	High	High	Low	High	Low	High	27.5
	Liu JCJ et al., 202	Low	Low	Low	Low	High	Low	Low	34.5
B_Stress									
	Chao M. et al., 2020	Low	Low	Low	Low	Uncertain	Low	Low	10.6
	Ruggieri S et al., 2020	Low	High	High	Low	High	Low	High	7.9
	Liu JCJ et al., 202	Low	Low	Low	Low	High	Low	Low	11
R_Anxiety									
	Chao M et al., 2020	Low	Low	Low	Low	Uncertain	Low	Low	29.1
	Kazan Kızılkurt O et al., 2020	Low	Low	High	Low	Uncertain	Low	Low	29.7
	Drouin M et al., 2020	Low	High	Low	Low	Uncertain	Low	Low	20.6
	Drouin M et al., 2020	Low	High	Low	Low	Uncertain	Low	Low	20.6
R_Stress									
	Chao M et al., 2020	Low	Low	Low	Low	Uncertain	Low	Low	34.2
	Hong W et al., 2020	Low	High	Low	Low	Uncertain	Low	Low	26.1
	Drouin M et al., 2020	Low	High	Low	Low	Uncertain	Low	Low	19.8
	Drouin M et al., 2020	Low	High	Low	Low	Uncertain	Low	Low	19.8

Supplementary material 4-1. Results for publication bias [a]

**Outcome**<br>Estimates	**Studies, n (participants)**	**Intercept [95% CI]**	**t-value**	**p-value**
**Anxiety symptoms**				
Odds ratio	6 (9,579)	-1.14 [-5.46-3.19]	-0.51	0.63
Regression coefficients	3 (2,376)	0.03 [-4.25-4.31]	0.01	0.99
Correlation coefficients	4 (2,483)	2.85 [-3.55-9.24]	0.87	0.48
**Depressive symptoms**				
Odds ratio	6 (13,241)	-1.14 [-5.46-3.19]	-0.51	0.63
Regression coefficients	3 (2,574)	2.53 [1.98-3.07]	9.07	0.07

CI- Confidence Interval

[a] Publication bias was tested with Egger's test

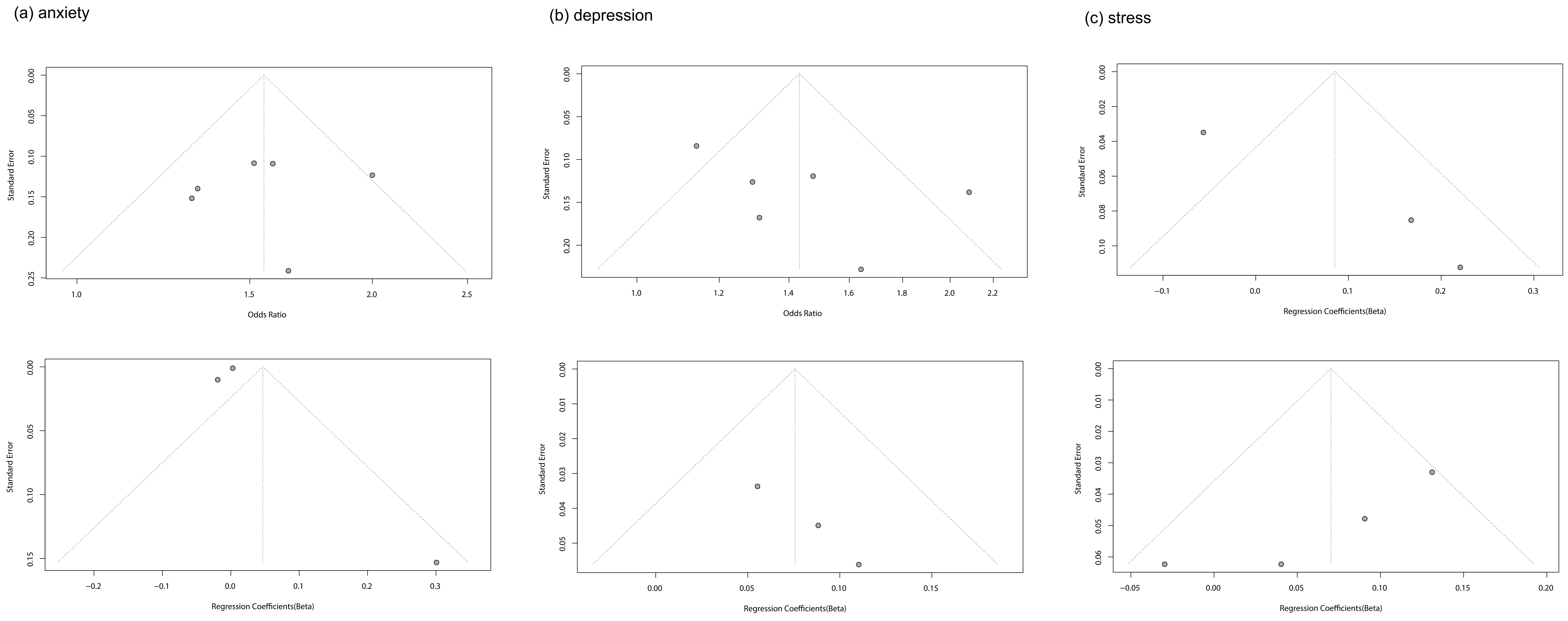

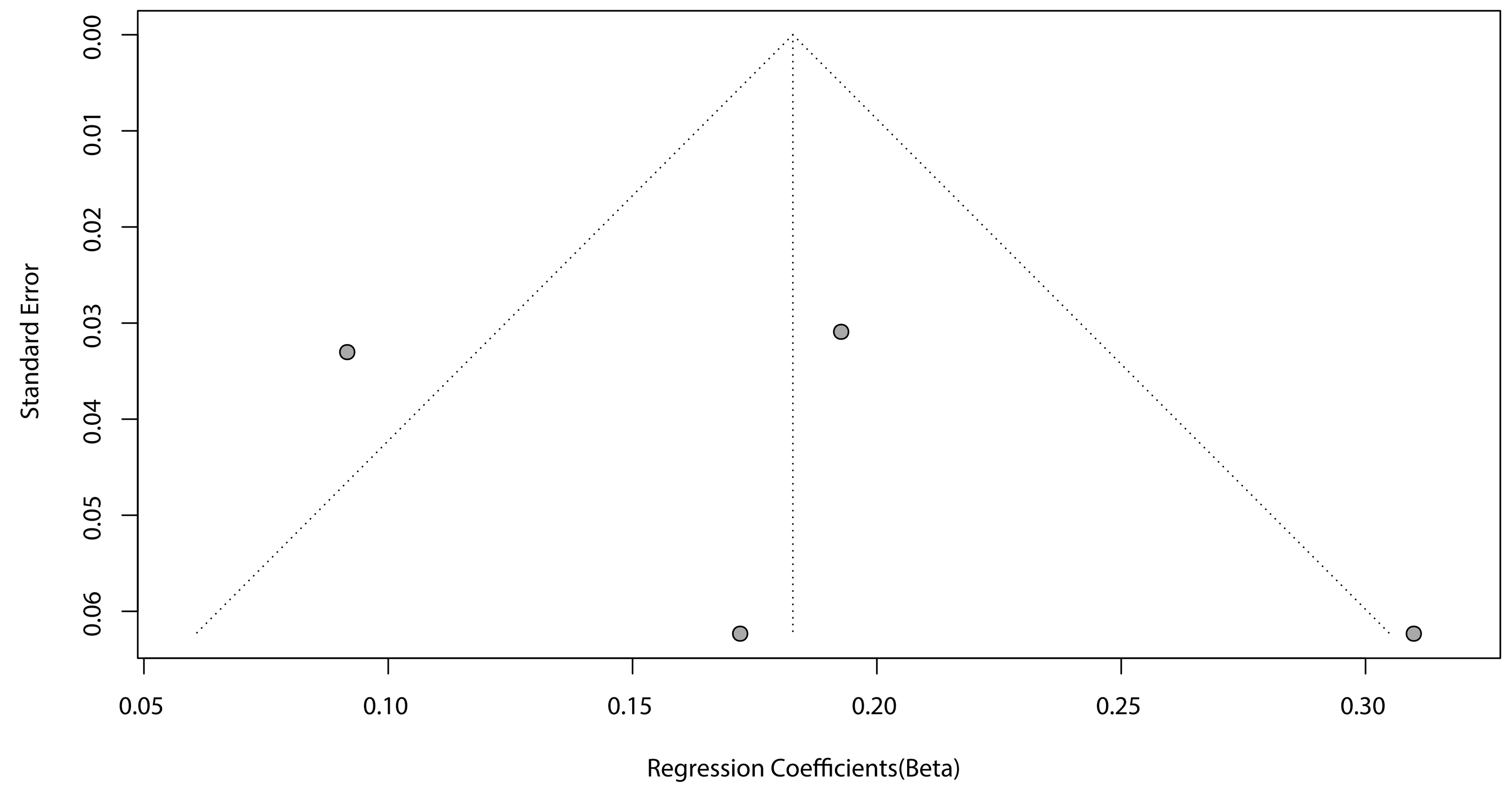

Supplementary Material. 4-2 Funnel plots for publication bias on relationship between social media exposure time and risk of various mental health symptoms in case-control studies. Each column represents funnel plots of (a) anxiety, (b) depression, and (c) stress.

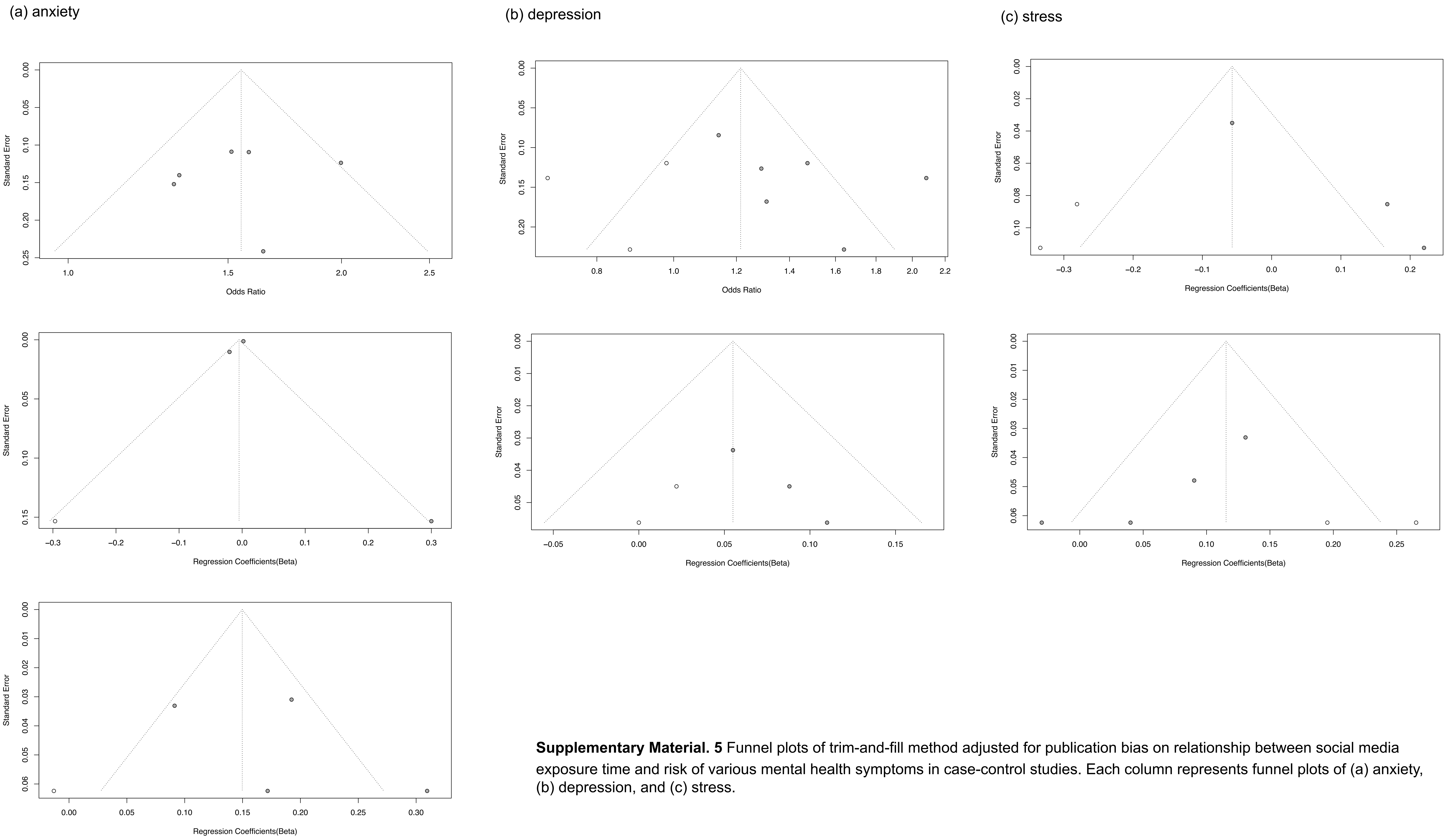

Supplementary Material. 5 Funnel plots of trim-and-fill method adjusted for publication bias on relationship between social media exposure time and risk of various mental health symptoms in case-control studies. Each column represents funnel plots of (a) anxiety, (b) depression, and (c) stress.

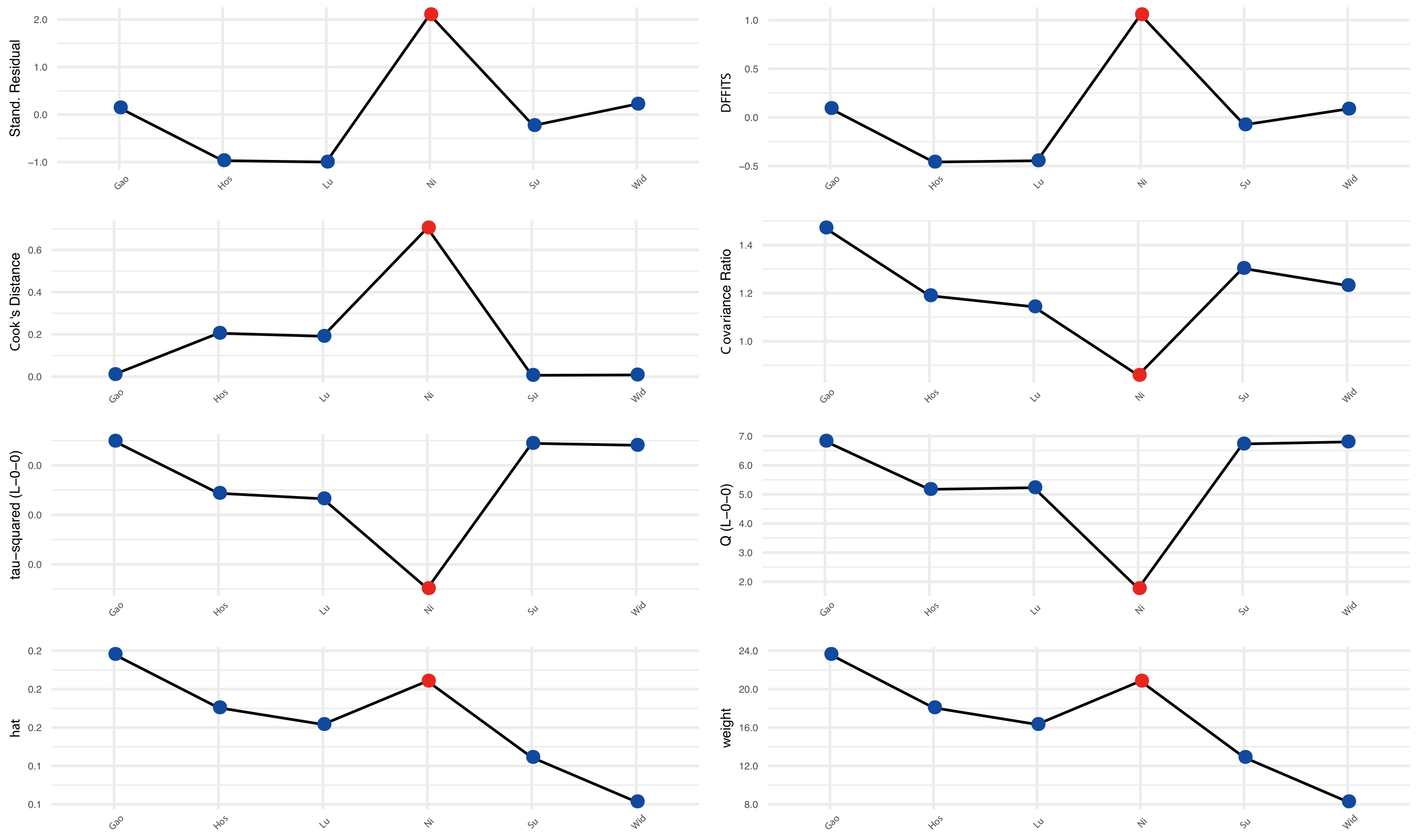

< Supplementary material 6-1 : influential analysis, Odds ratio, Anxiety >

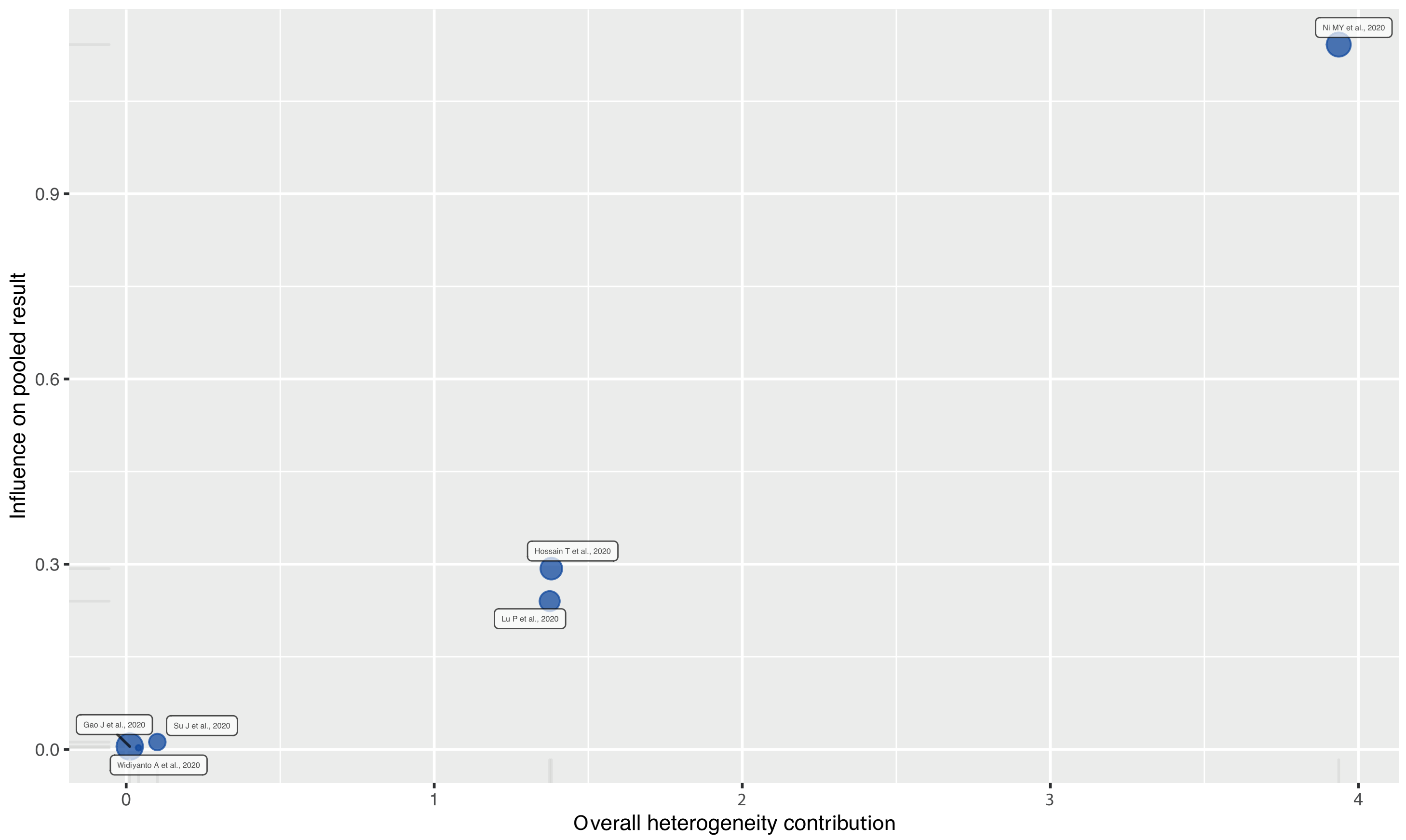

< Supplementary material 6-2 : Baujat plot, Odds ratio, Anxiety >

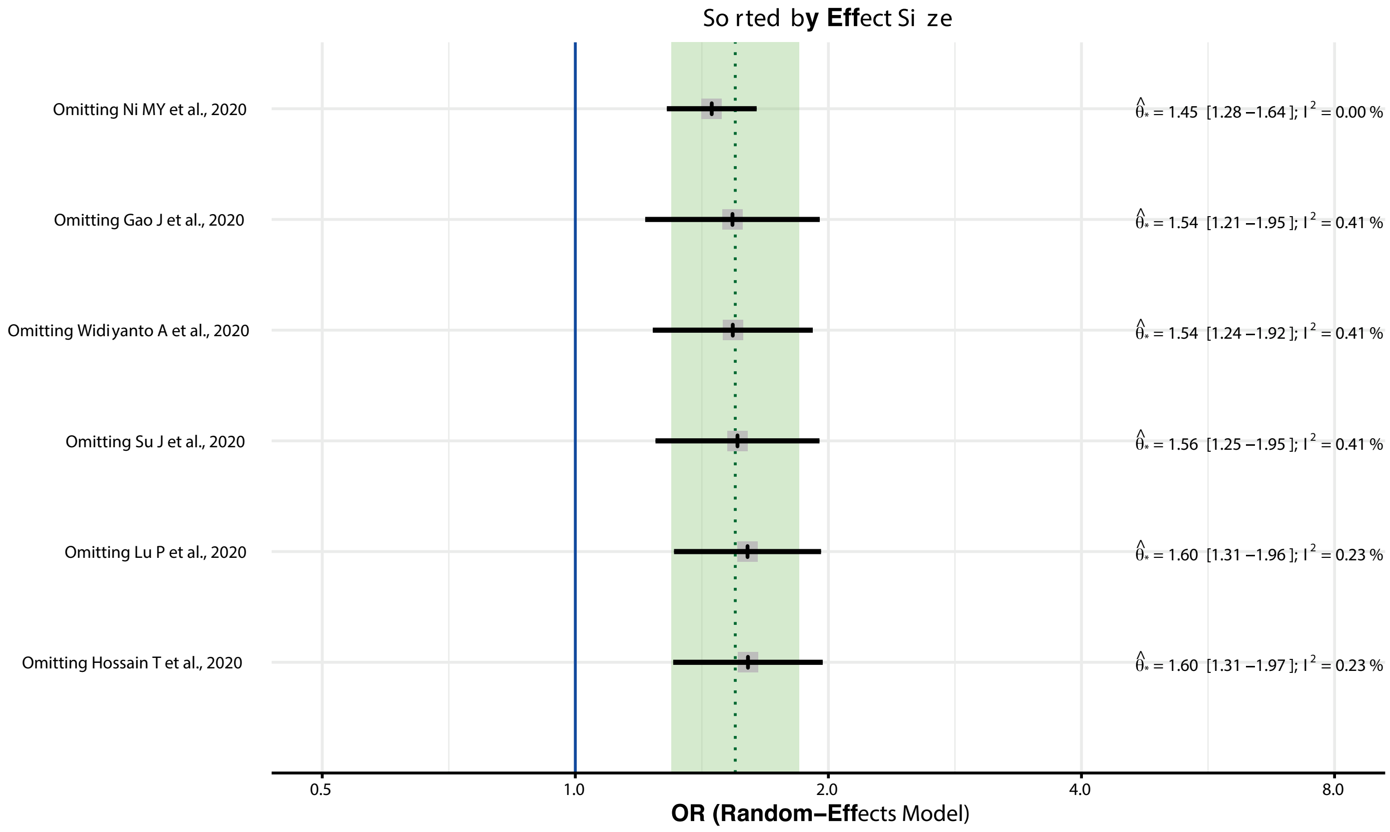

< Supplementary material 6-3 : Influential analysis: sorted by effect size, Odds ratio, Anxiety >

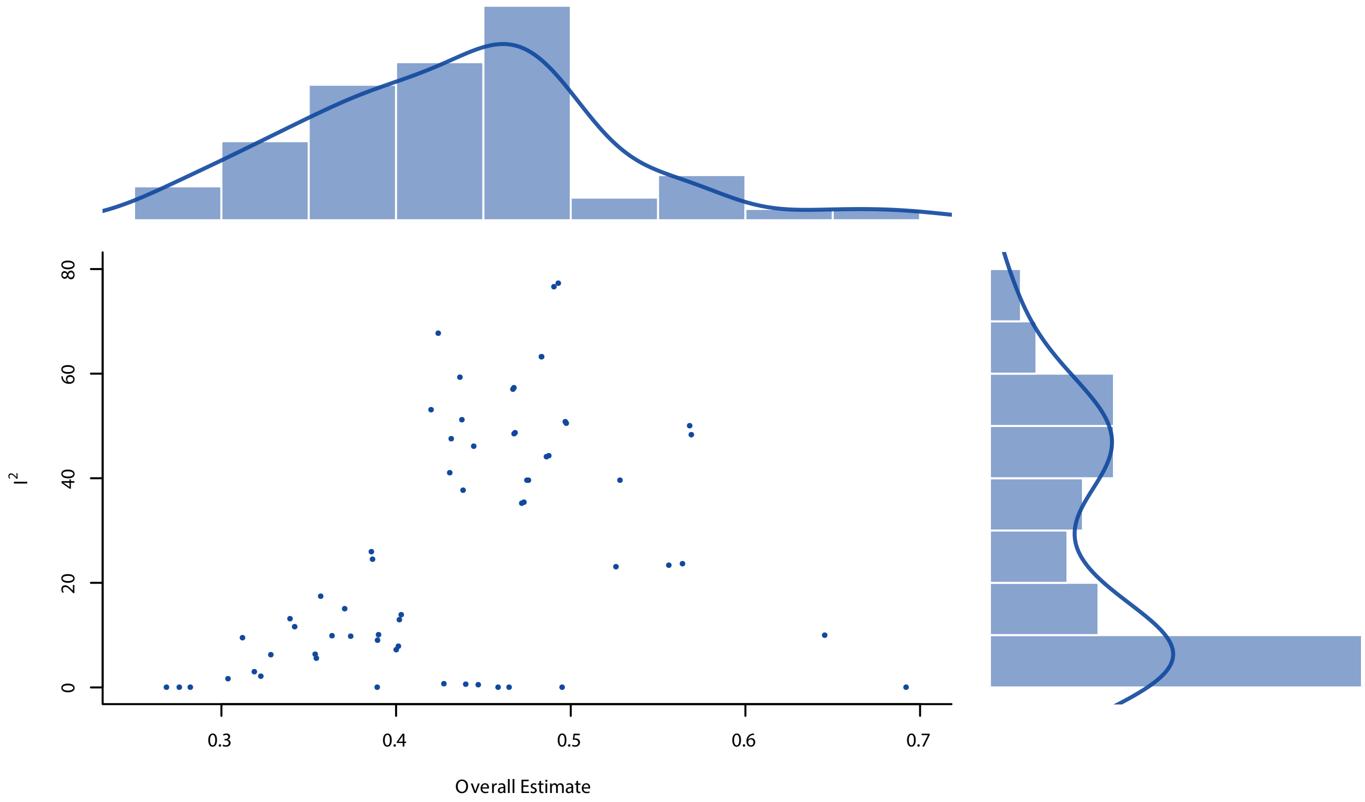

< Supplementary material 6-4 : GOSH plot, Odds ratio, Anxiety >

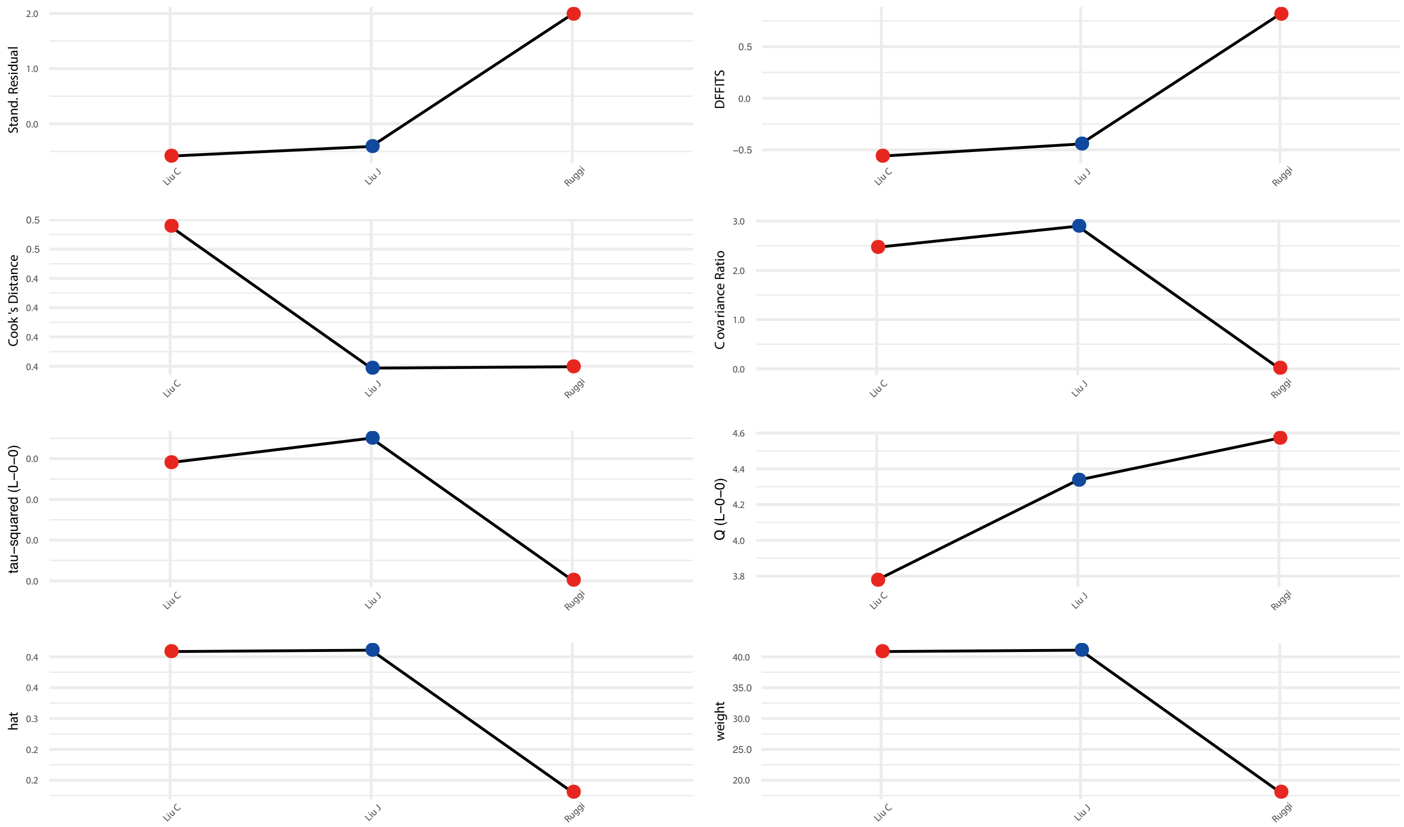

< Supplementary material 6-5: influential analysis, Beta, Anxiety>

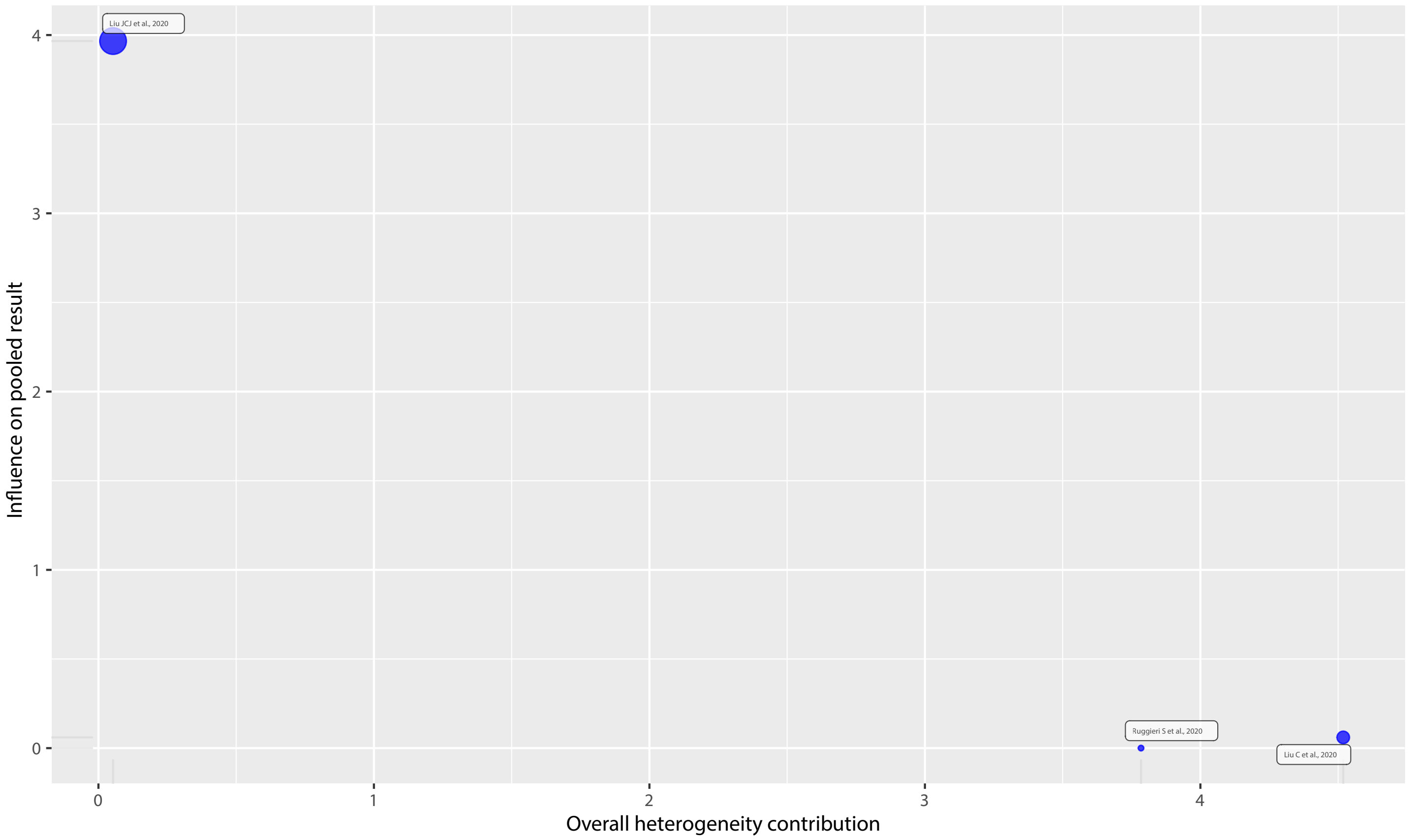

< Supplementary material 6-6: Baujat plot, Beta, Anxiety >

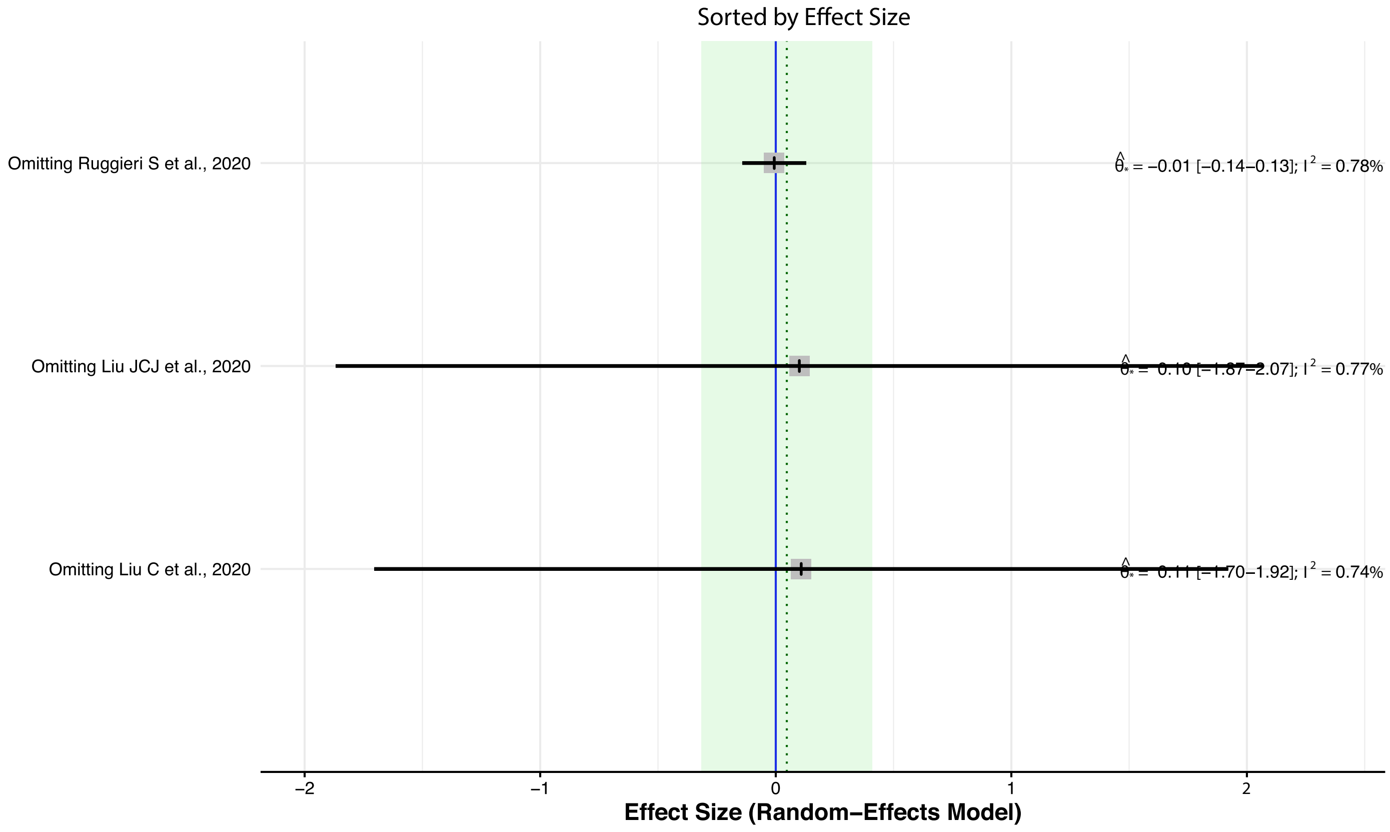

< Supplementary material 6-7 : Influential analysis: sorted by effect size, Beta, Anxiety >

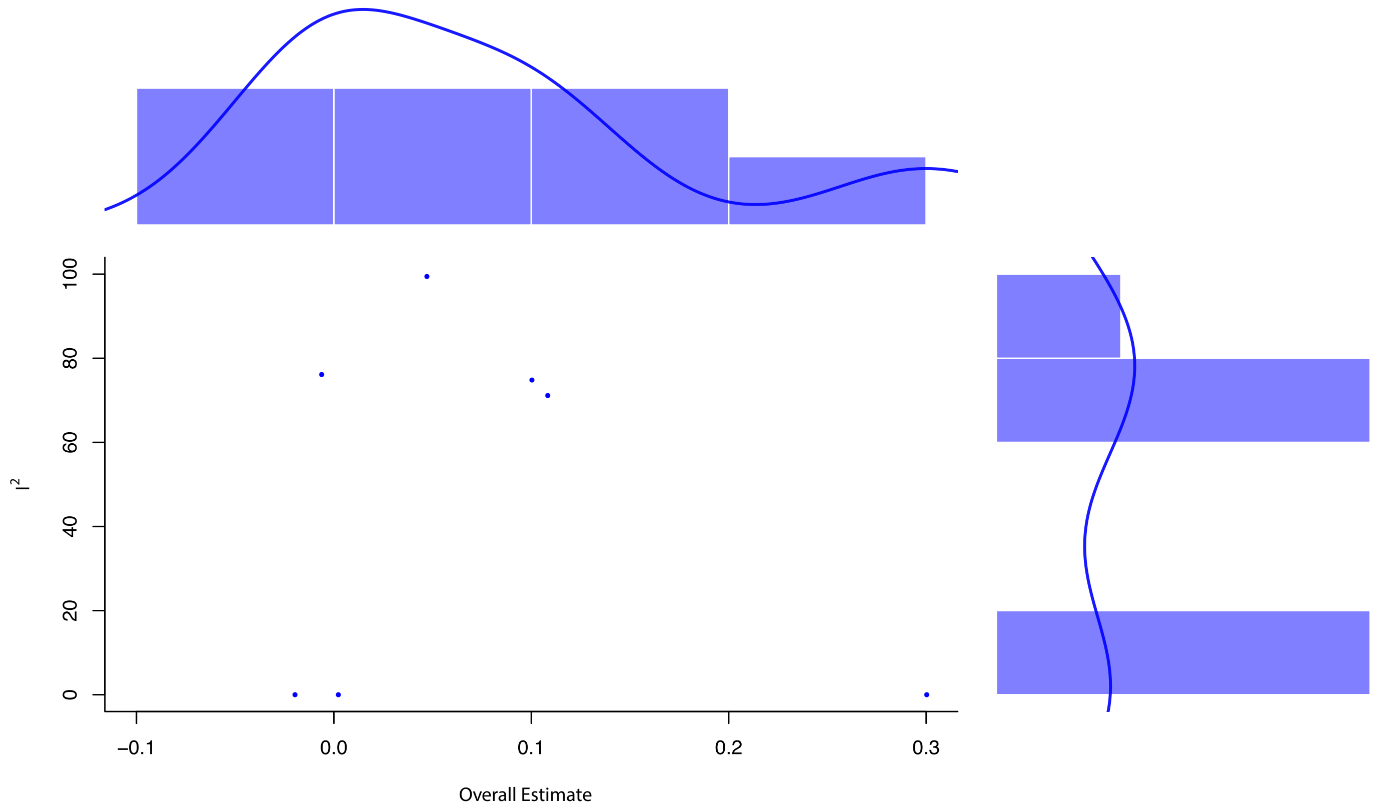

< Supplementary material 6-8: GOSH plot, Beta, Anxiety >

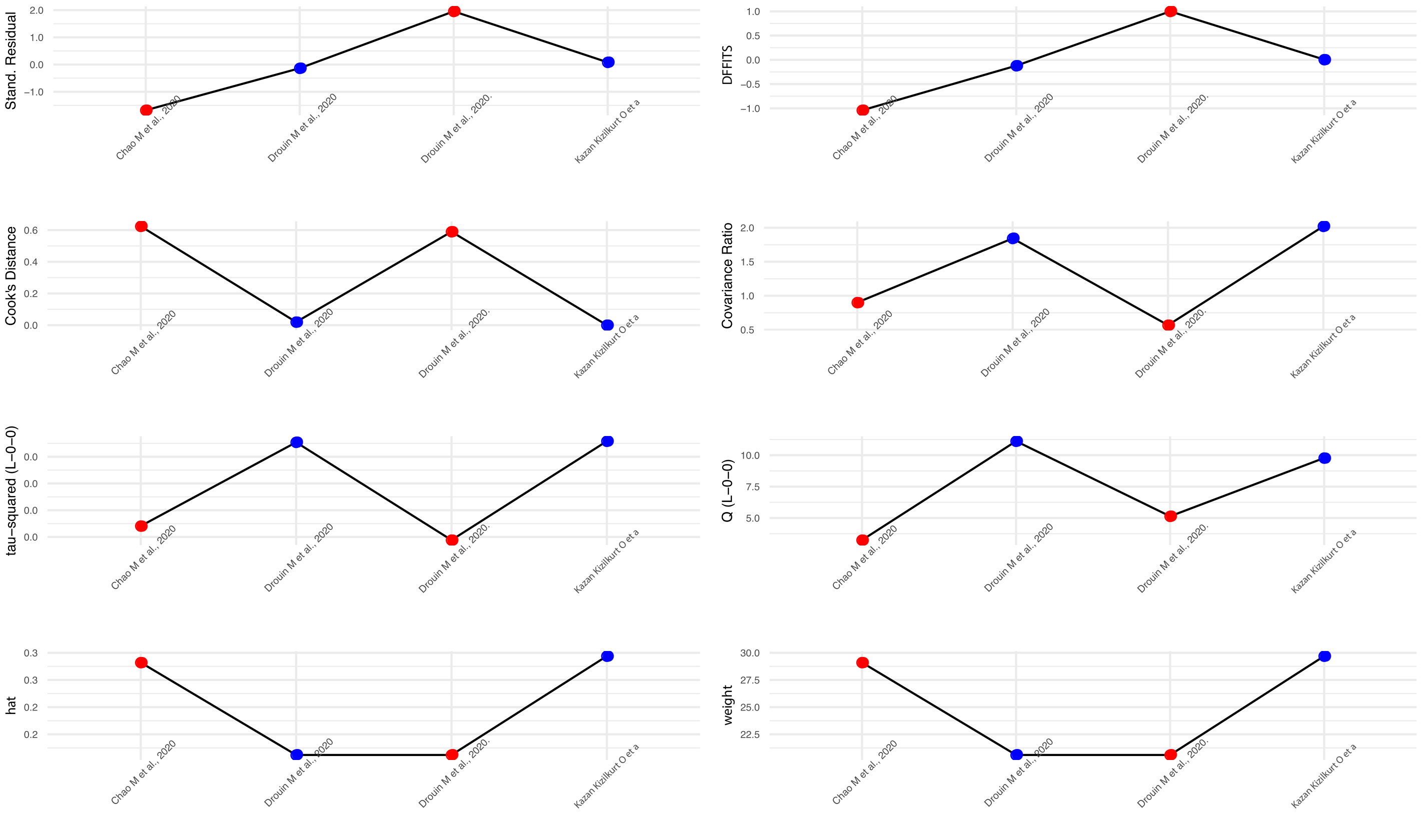

< Supplementary material 6-9 : influential analysis, correlation, Anxiety >

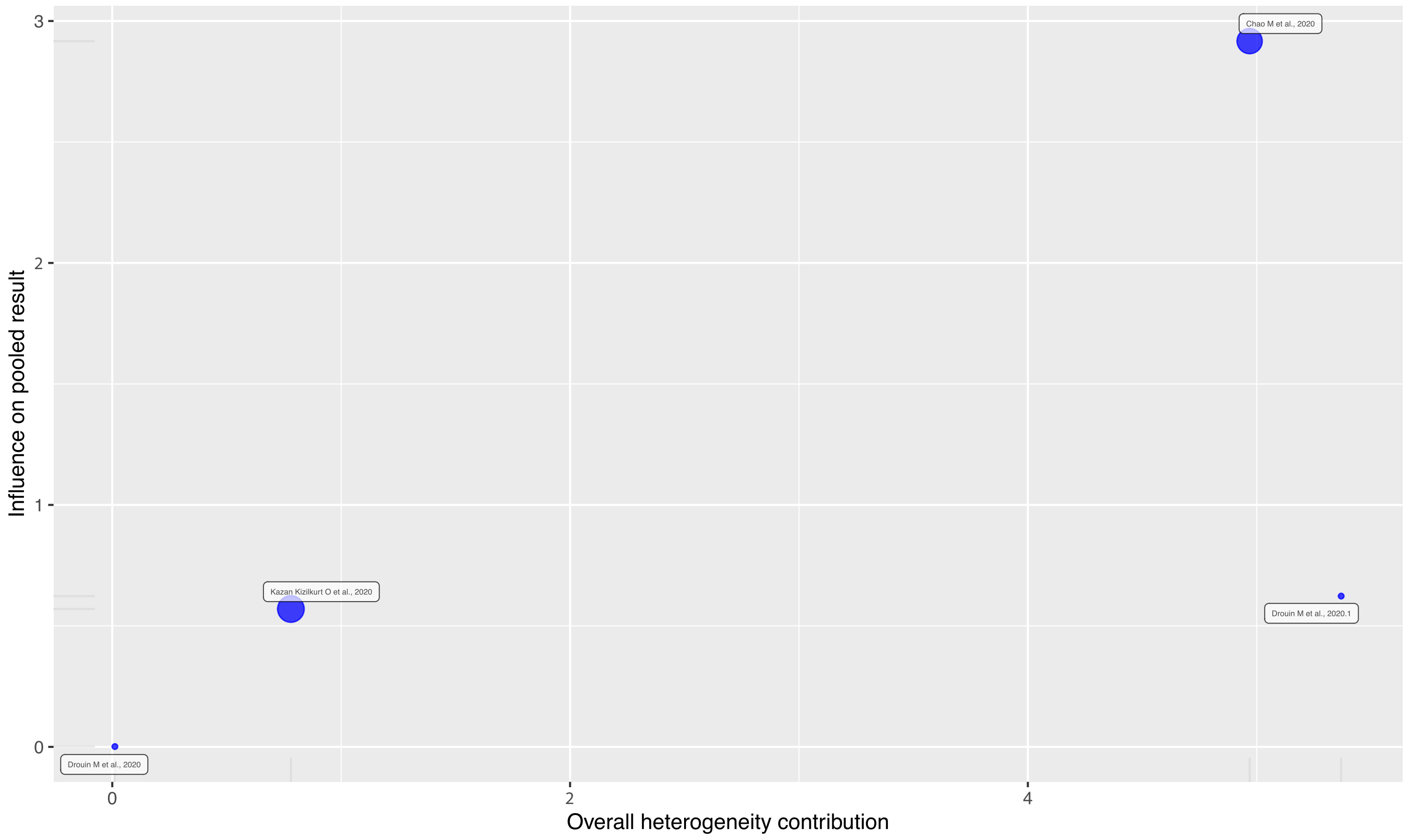

< Supplementary material 6-10: Baujat plot, correlation, Anxiety >

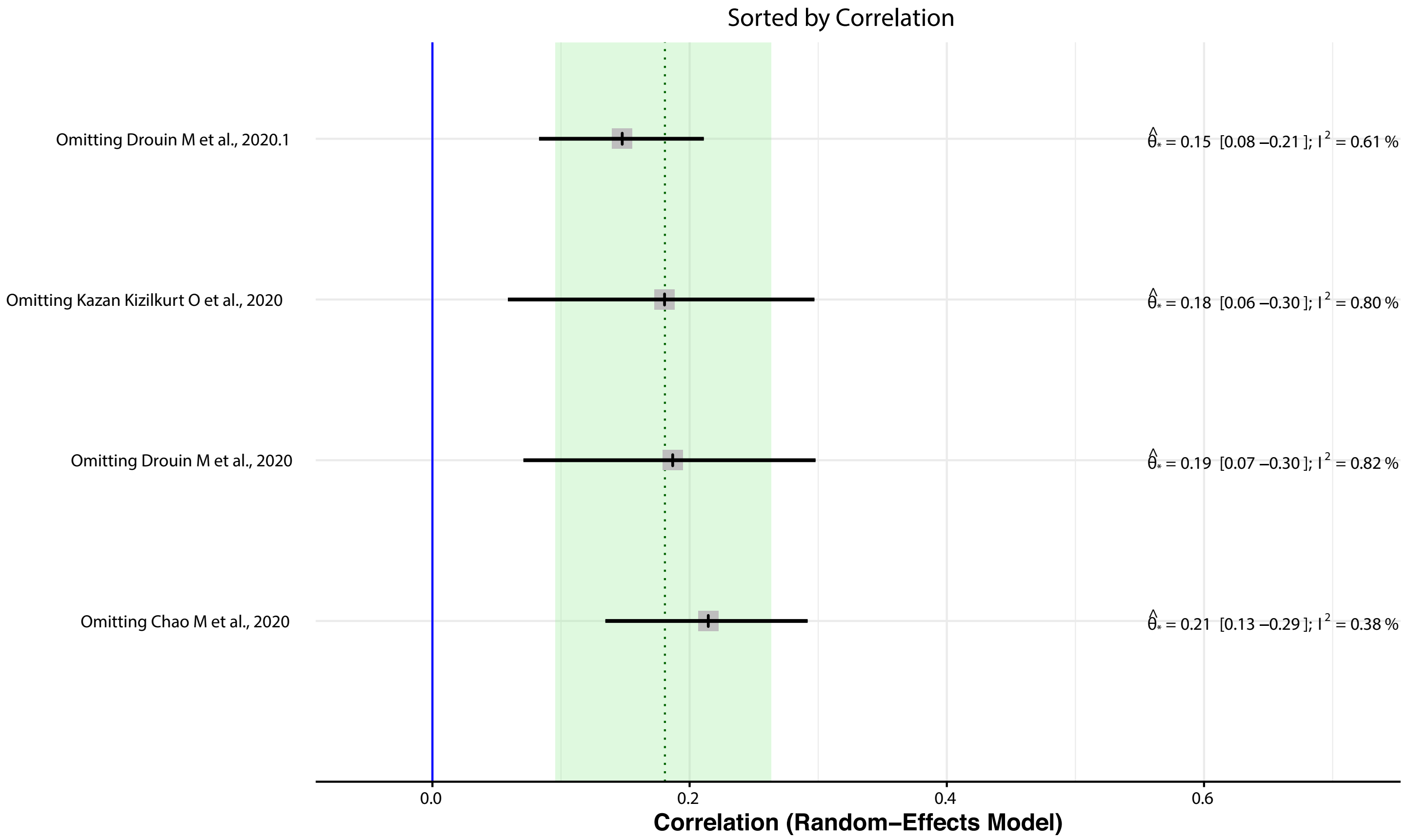

< Supplementary material 6-11: Influential analysis: sorted by effect size, correlation, Anxiety >

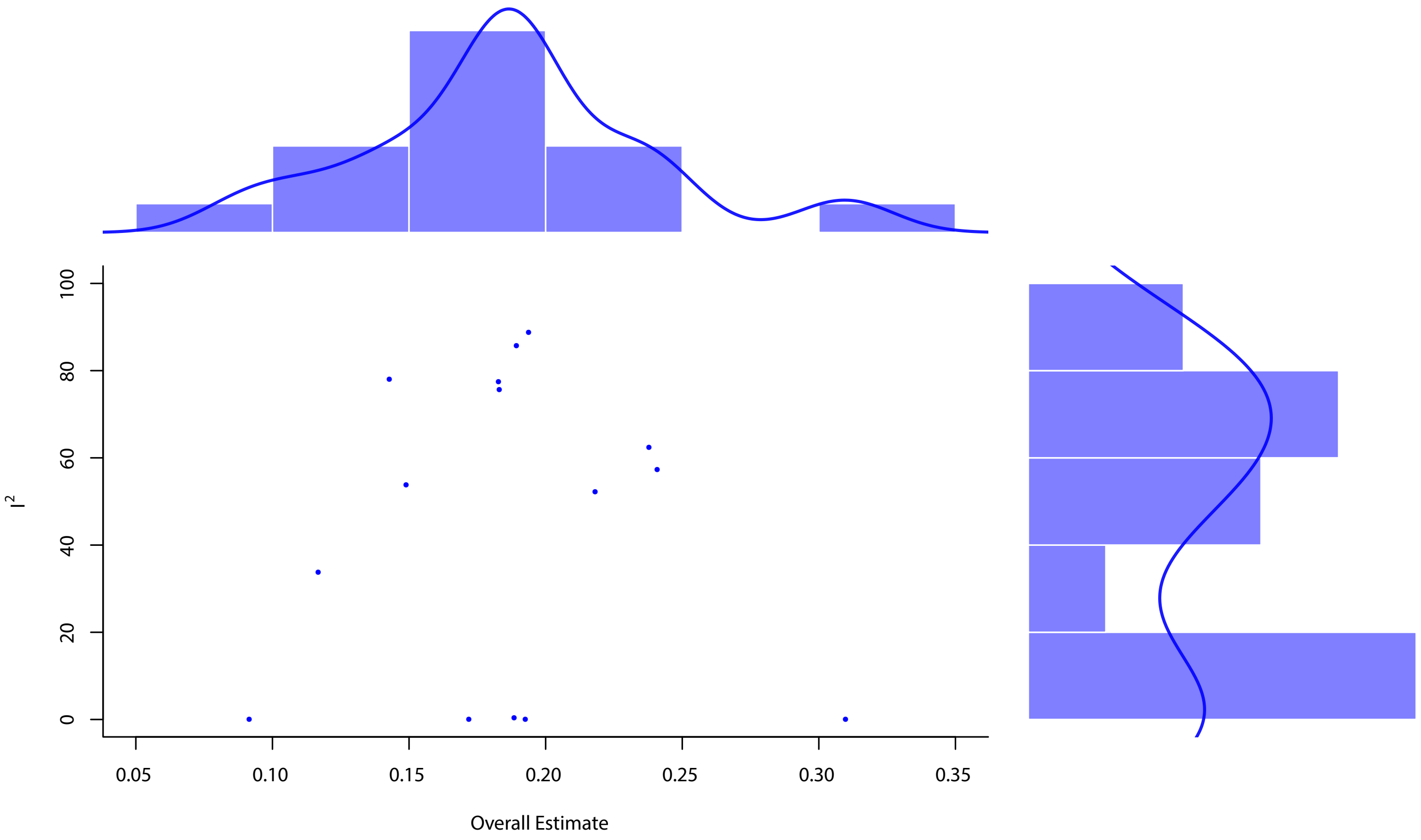

< Supplementary material 6-12: GOSH plot, correlation, Anxiety >

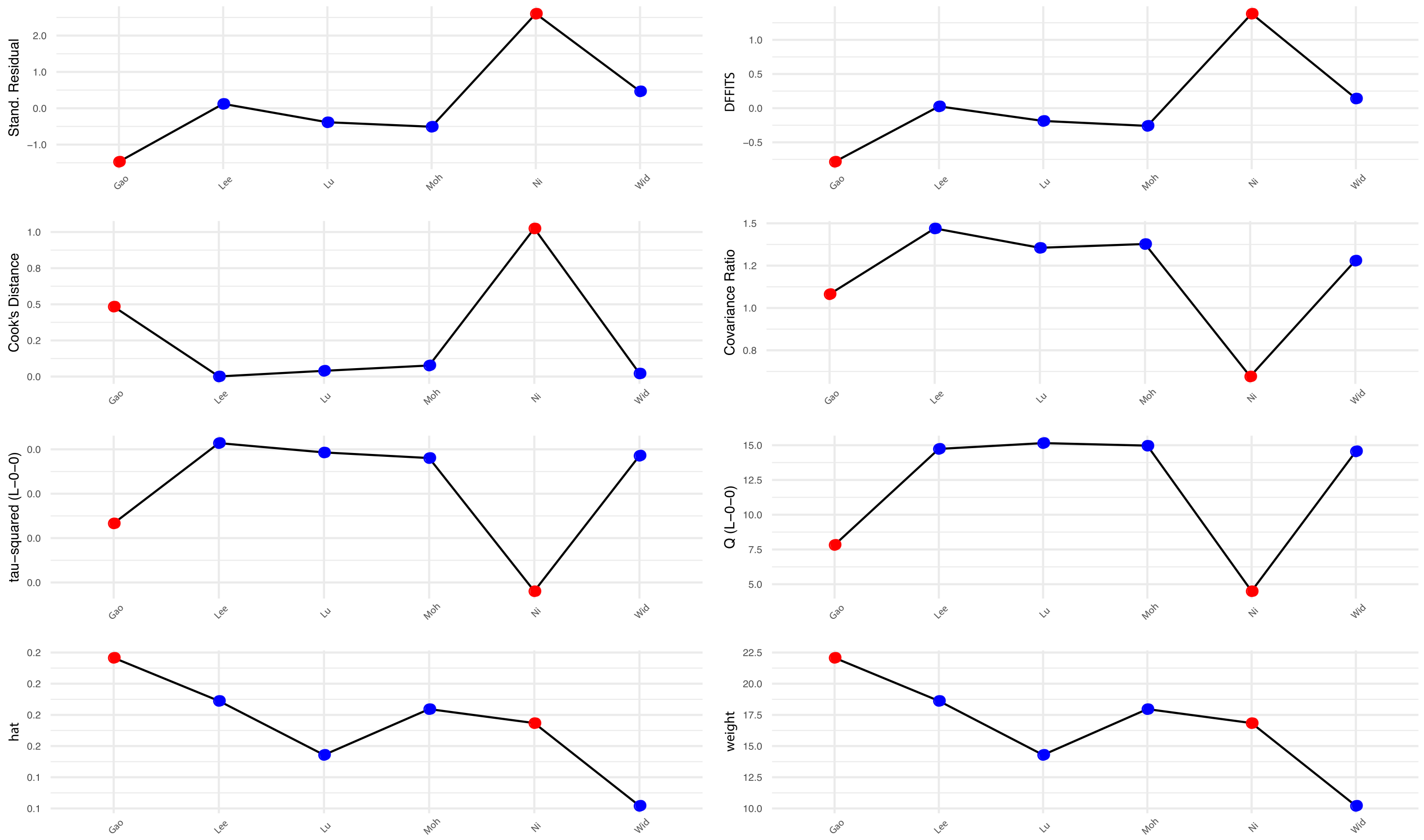

< Supplementary material 6-13: influential analysis, odds ratio, Depression >

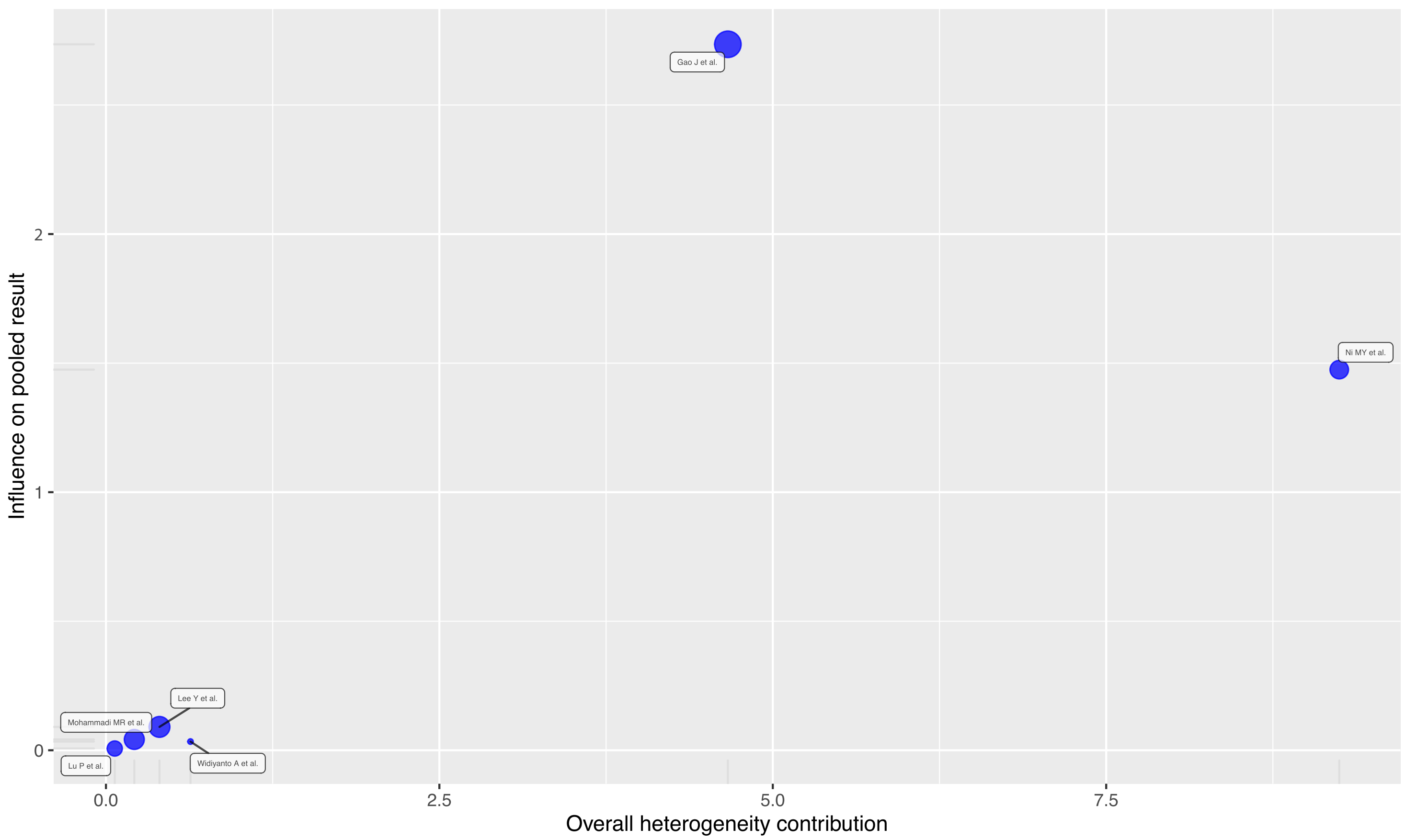

< Supplementary material 6-14: Baujat plot, odds ratio, Depression >

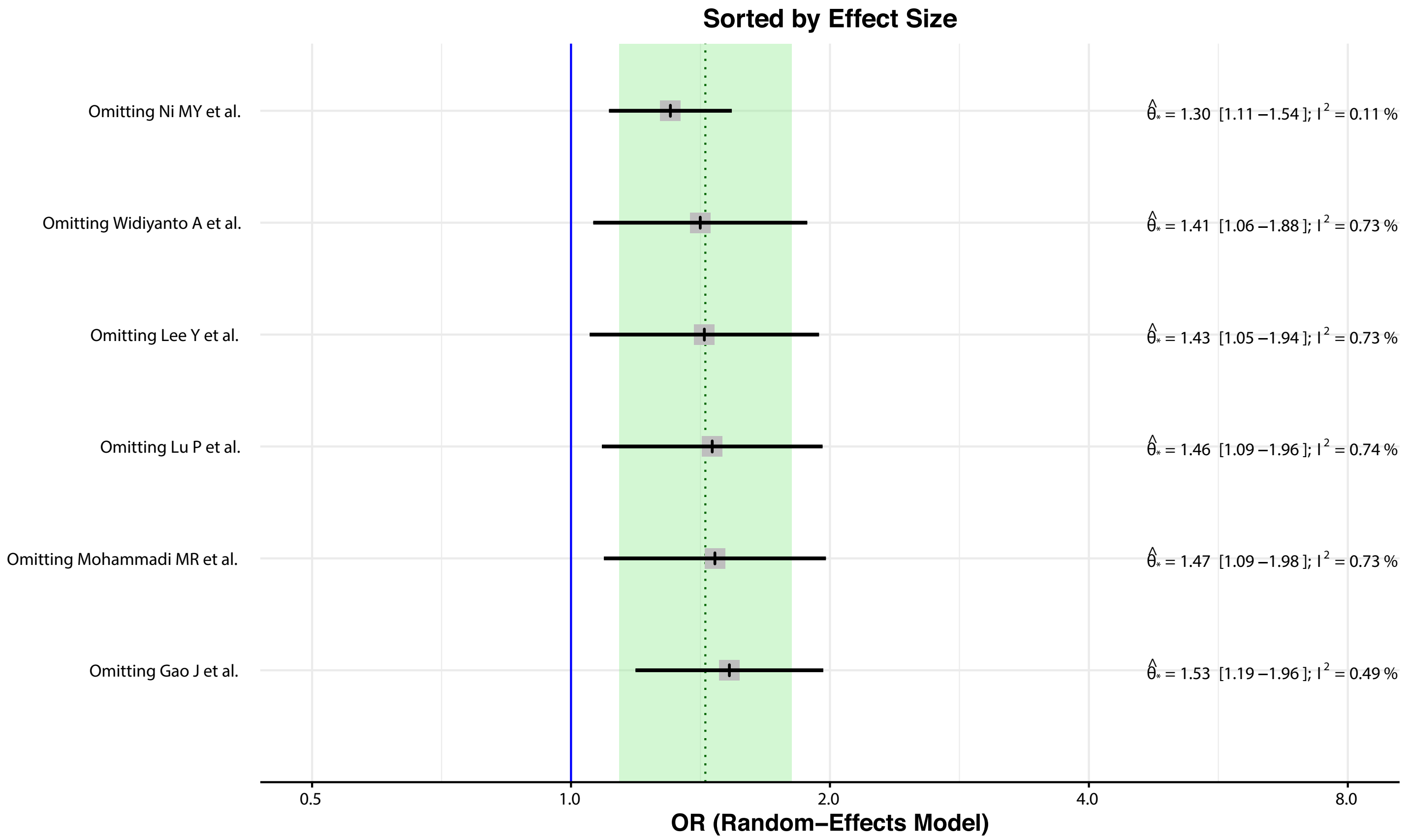

< Supplementary material 6-15: Influential analysis: sorted by effect size, odds ratio, Depression >

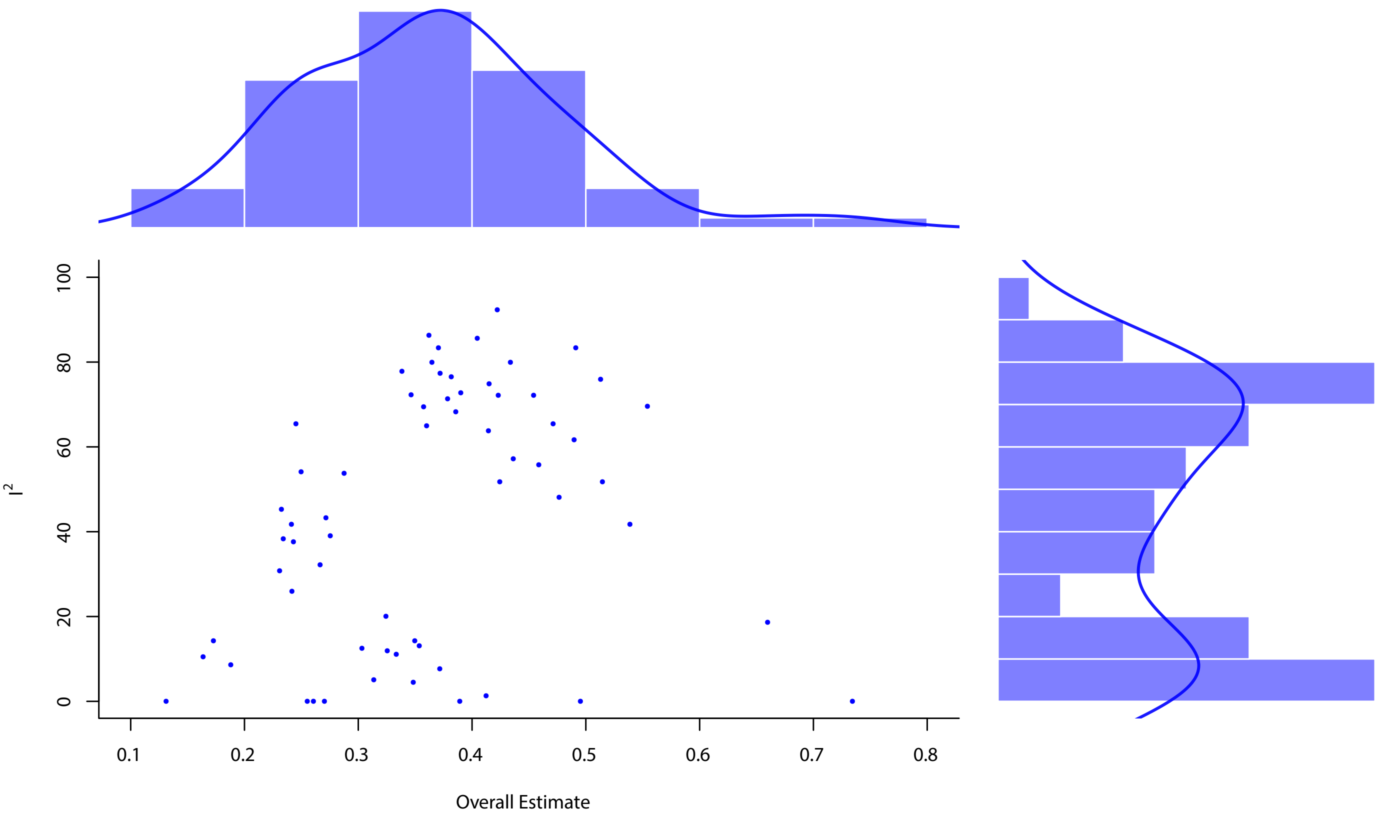

< Supplementary material 6-16: GOSH plot, Odds ratio, Depression >

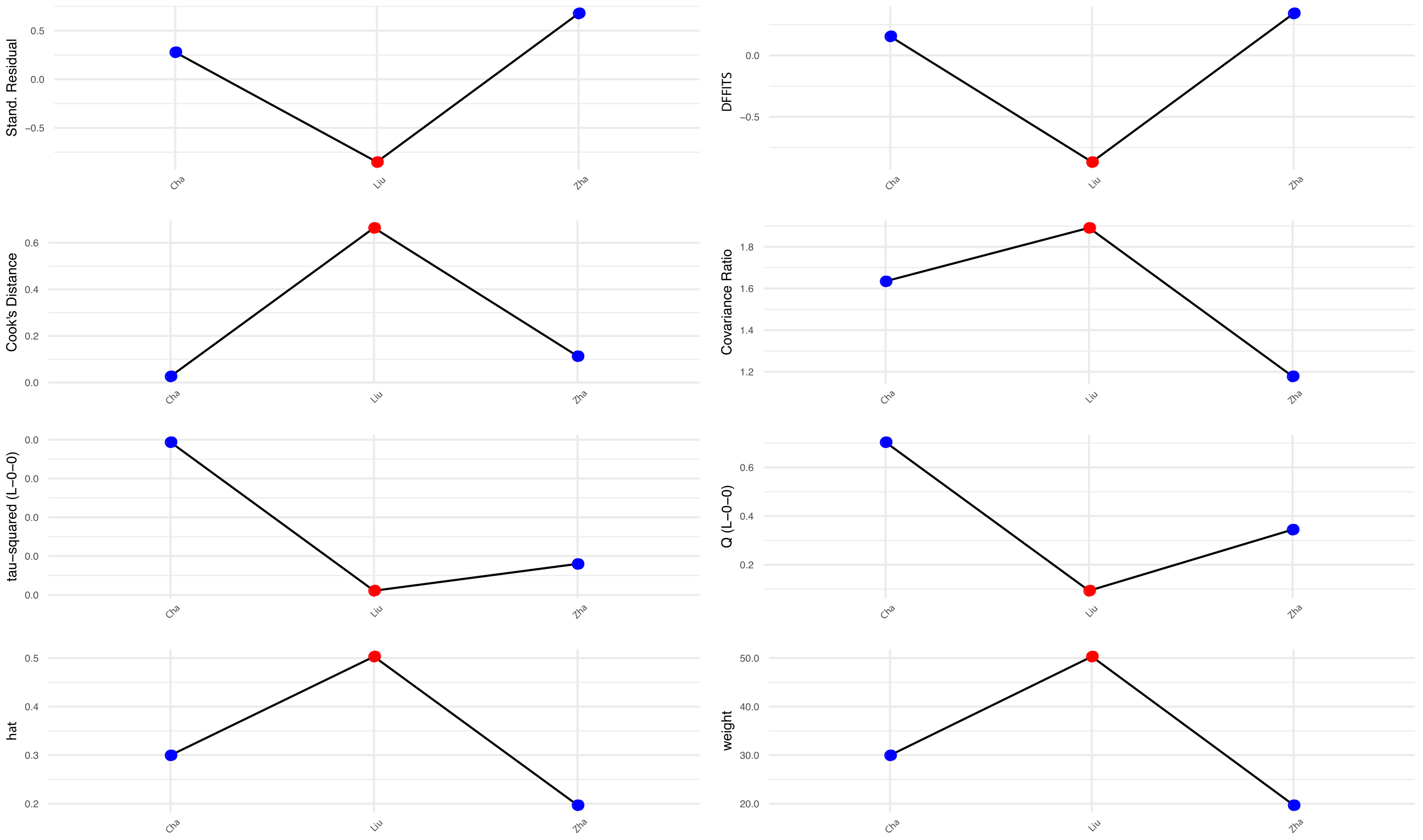

< Supplementary material 6-17: influential analysis, Beta, Depression >

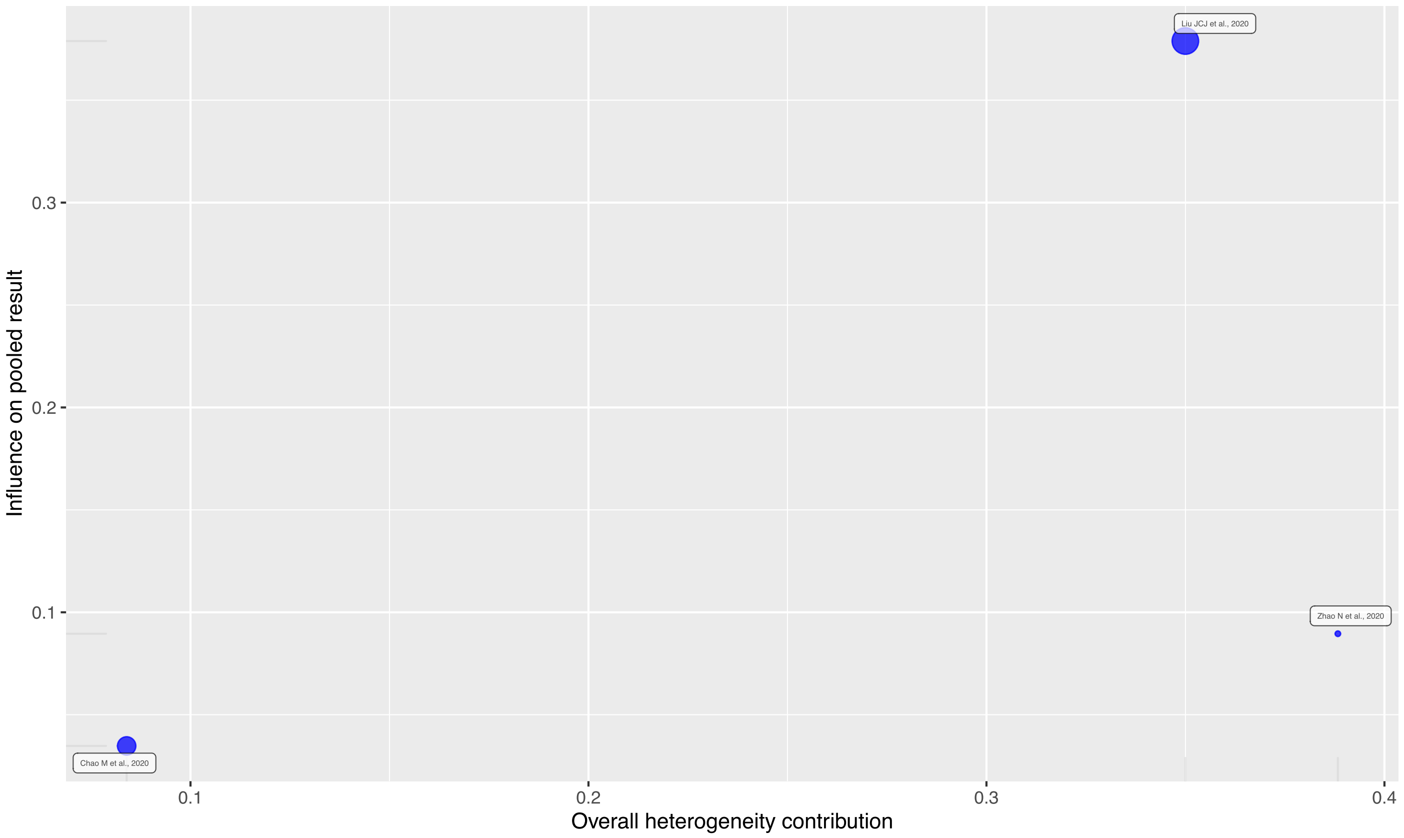

< Supplementary material 6-18: Baujat plot, Beta, Depression >

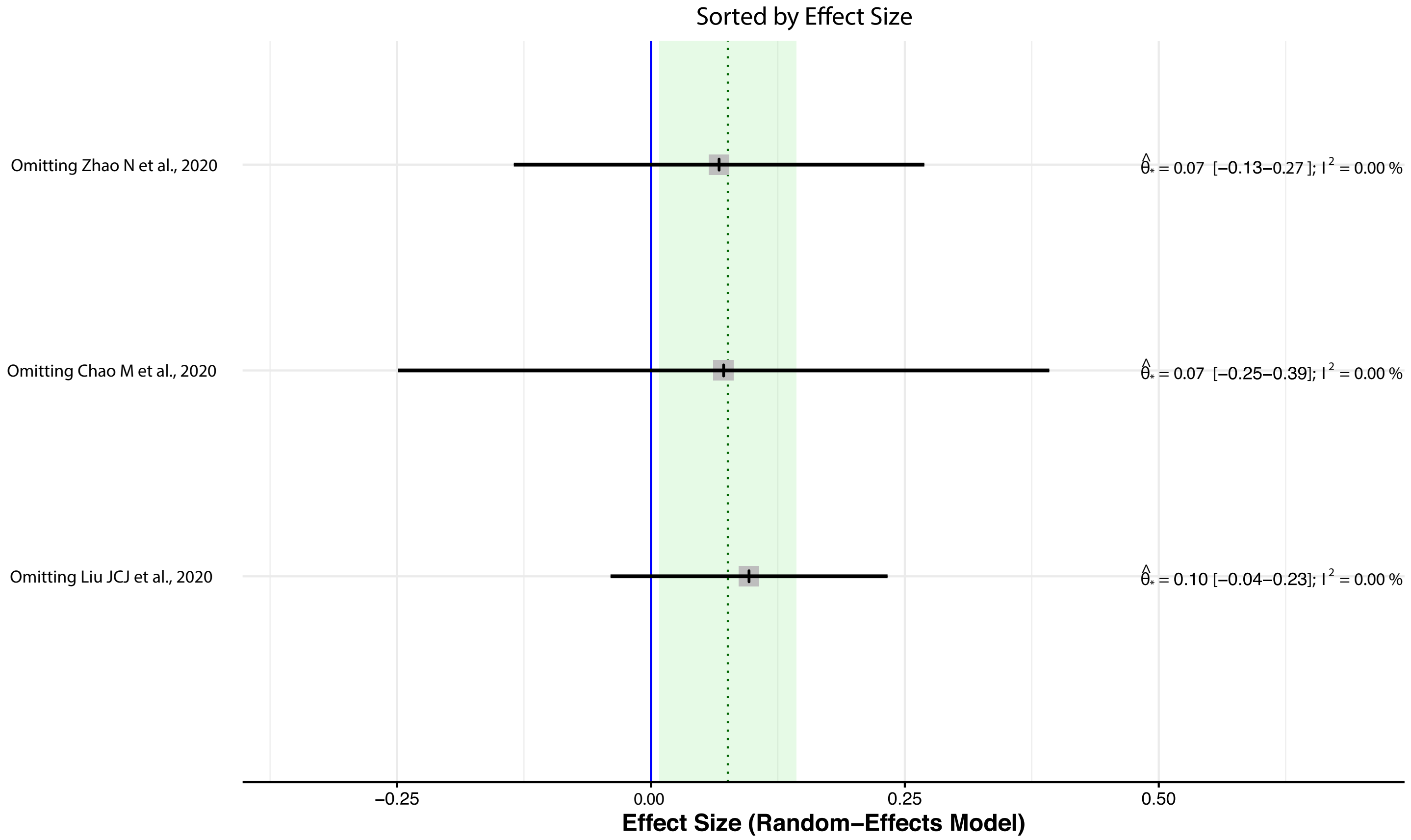

< Supplementary material 6-19: Influential analysis: sorted by effect size, Beta, Depression >

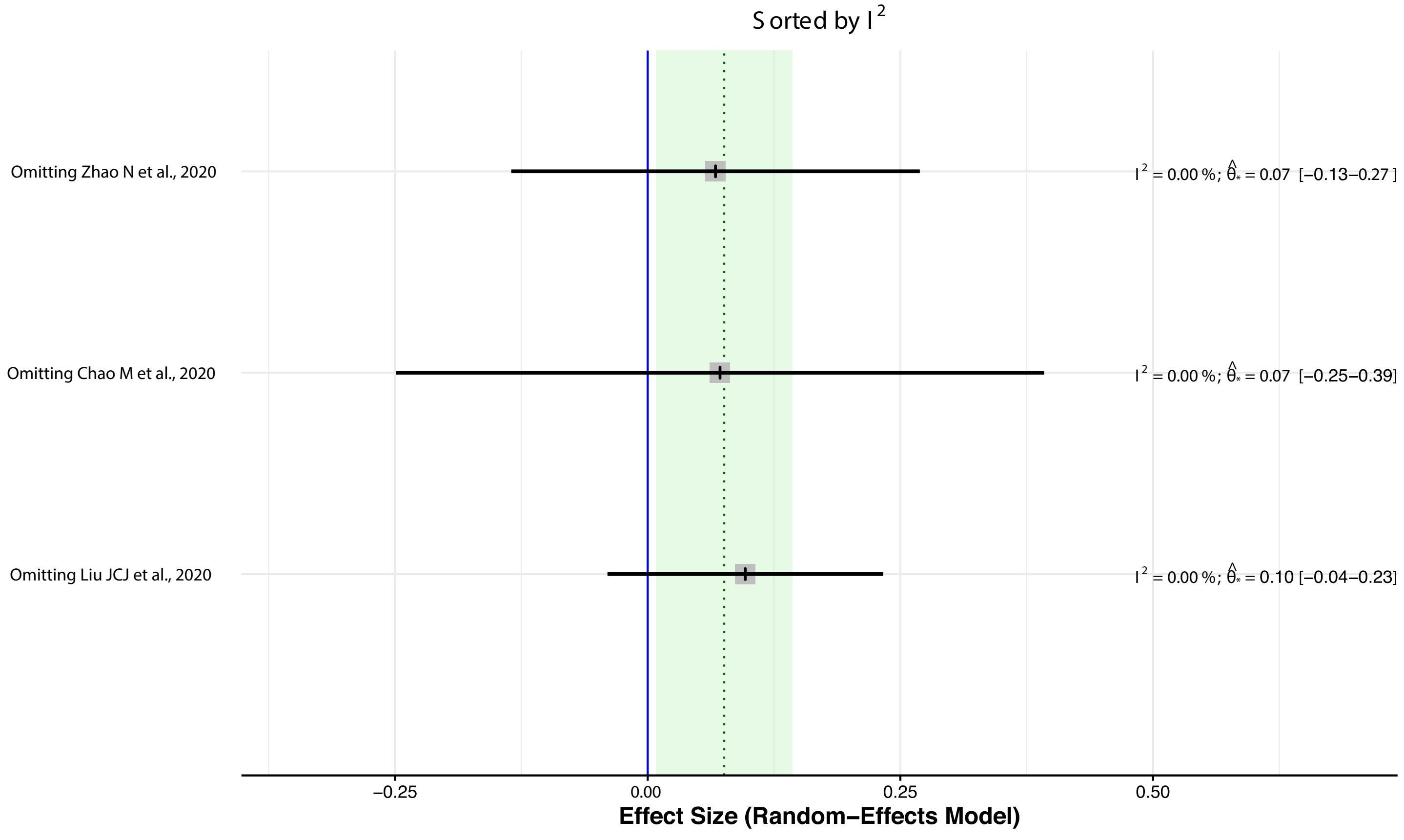

< Supplementary material 6-20: Influential analysis: sorted by I^2, Beta, Depression >

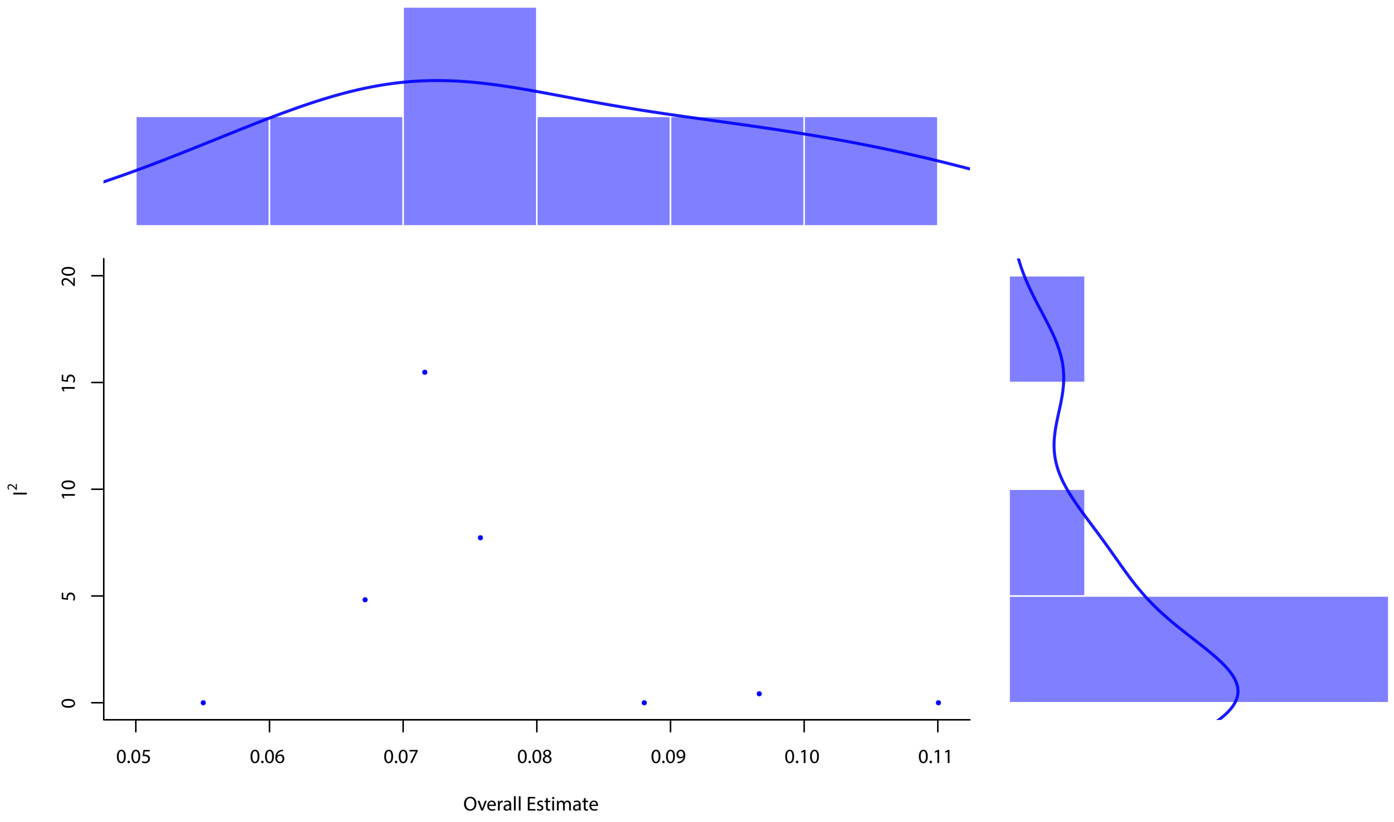

< Supplementary material 6-21: GOSH plot, Beta, Depresion >

Supplementary material 7. Results for statistical inter- study heterogeneity assessment

Outcome<br>Estimates	Overall effect size<br><br>ES (I^2 %)	Outlier detection<br><br>ES (I^2 %)	Influential Analysis<br>ES (I^2 %)	GOSH analysis<br><br>ES (I^2 %)
Anxiety symptoms				
Odds ratio	1.55 (26.94%)	1.55 (26.94%)	1.45 (0.00%)	1.55 (26.94%)
Beta	0.05 (76.07%)	0.05 (76.07%)	0.11 (73.50%)	0.05 (76.07%)
Pearson's r	0.18 (73.04%)	0.18 (73.04%)	0.22 (38.34%)	0.18 (73.04%)
Depressive symptoms				
Odds ratio	1.43 (67.15%)	1.43 (67.15%)	1.30 (11.14%)	1.43 (67.15%)
Beta	0.07 (0.00%)	0.07 (0.00%)	0.10 (0.00%)	0.07 (0.00%)

CI- Confidence Interval; ES- Effect size; GOSH– a Graphical Display of Study Heterogeneity.
