# Supplementary material for: Social media use and mental health during the COVID-19 pandemic in young adults: a meta-analysis of 14 cross-sectional studies
Source: BMC Public Health. 2022 May 17;22:995. doi: 10.1186/s12889-022-13409-0 (PMC9112239; doi:10.1186/s12889-022-13409-0)
Supplement: Supplementary file 2 — Additional file 2. [file 12889_2022_13409_MOESM2_ESM.pdf]

**Supplementary material 8. List of retrieved papers sorted by given type of estimates and research outcomes**			
**Study outcome**	**Given estimates in each study**		
	**Odds ratio (N=12)**	**regression coefficients (N=6)**	**Pearson's r (N=4)**
**Anxiety (N=13)**	1. Gao J et al. 2020	7. Liu JCJ et al. 2020	10. Chao M et al. 2020
	2. Lu P et al. 2020	8. Chao M et al. 2020	11. Kazan Kızılkurt O et al. 2020
	3. Widiyanto A et al. 2020	9. Ruggieri S et al. 2020	12. Drouin M et al. 2020
	4. Hossain T et al. 20		13. Drouin M et al. 2020
	5. Ni MY et al. 2020		
	6. Su J et al. 2020		
**Depression (N=9)**	1. Gao J et al. 2020	7. Liu JCJ et al. 2020	
	2. Lu P et al. 2020	8. Chao M et al. 2020	
	3. Mohammadi MR et al. 2020	9. Ruggieri S et al. 2020	
	4. Widiyanto A et al. 2020		
	5. Lee Y et al. 2020		
	6. Ni MY et al. 2020		
Duplicate studies are shaded.			
